# Morphine-responsive neurons that regulate mechanical antinociception

**DOI:** 10.1126/science.ado6593

**Published:** 2024-08-30

**Authors:** Michael P. Fatt, Ming-Dong Zhang, Jussi Kupari, Müge Altınkök, Yunting Yang, Yizhou Hu, Per Svenningsson, Patrik Ernfors

**Affiliations:** 1 Division of Molecular Neurobiology, Department of Medical Biochemistry and Biophysics, Karolinska Institutet, 171 65 Stockholm, Sweden; 2 Division of Neuro, Department of Clinical Neuroscience, Karolinska Institutet, 171 77 Stockholm, Sweden

## Abstract

Opioids are widely used effective analgesics to manage severe acute and chronic pain, although they have recently come under scrutiny because of epidemic levels of abuse. While these compounds act on numerous central and peripheral pain pathways, the neuroanatomical substrate for opioid analgesia is not fully understood. By means of single cell transcriptomics and manipulation of morphine-responsive neurons, we have identified an ensemble of neurons in the rostral ventromedial medulla (RVM) that regulates mechanical nociception in mice. Among these, forced activation or silencing of excitatory RVM^BDNF^ projection neurons mimicked or completely reversed morphine-induced mechanical antinociception, respectively, via a brain-derived neurotrophic factor (BDNF)/tropomyosin receptor kinase B (TrkB)-dependent mechanism and activation of inhibitory spinal galanin-positive neurons. Our results reveal a specific RVM-spinal circuit that scales mechanical nociception whose function confers the antinociceptive properties of morphine.

Opioids have been used for medicinal and recreational purposes for millennia. Opioids such as morphine remain effective treatments for managing pain and for improving physical function (1). However, overprescription and self-medication can lead to addiction, respiratory depression, and overdose-induced death. These concerns highlight the importance of understanding the neural basis for opioid antinociception. The rostral ventromedial medulla (RVM) is the final common output node of the complex, brain-spanning network that affects the experience of pain (2). Lesions of the RVM abolish morphine analgesia, whereas electrical stimulation induces powerful antinociception (3, 4). The analgesic effects of electrical stimulation as well as morphine depend on axonal tracts projecting from the RVM to the spinal cord (5). Spinal cord-projecting neurons terminate in the dorsal horn where nociceptive processing is affected, resulting in accentuation or attenuation of withdrawal reflexes. These spinal projections also control the processing of ascending transmission, thereby influencing affective and perceptive dimensions of pain (6). Depending on the strength of electrical stimulation in the RVM, nociception is either facilitated or inhibited (7, 8). Within the RVM, serotonergic (9), γ-aminobutyric acid-mediated [GABAergic (10–12)], and perhaps also glutamatergic neurons can modulate pain (13). Pain stimuli are associated with heightened activity of some RVM neurons and lowered activity of others [so called ON and OFF cells, (14, 15)]. In addition, µ-opioid receptors important for opioid analgesia are present in virtually all neural substrates in the brain contributing to the experience of pain, and consistently there is experimental evidence for the involvement of spinal as well as multiple supraspinal sites in analgesia (1, 16). Thus, the relative contribution of descending inhibition on ascending transmission executed by the RVM and the mechanisms involved have remained important questions for understanding opioid antinociception.

## A morphine-responsive antinociceptive ensemble in the RVM

**Fig 1:**
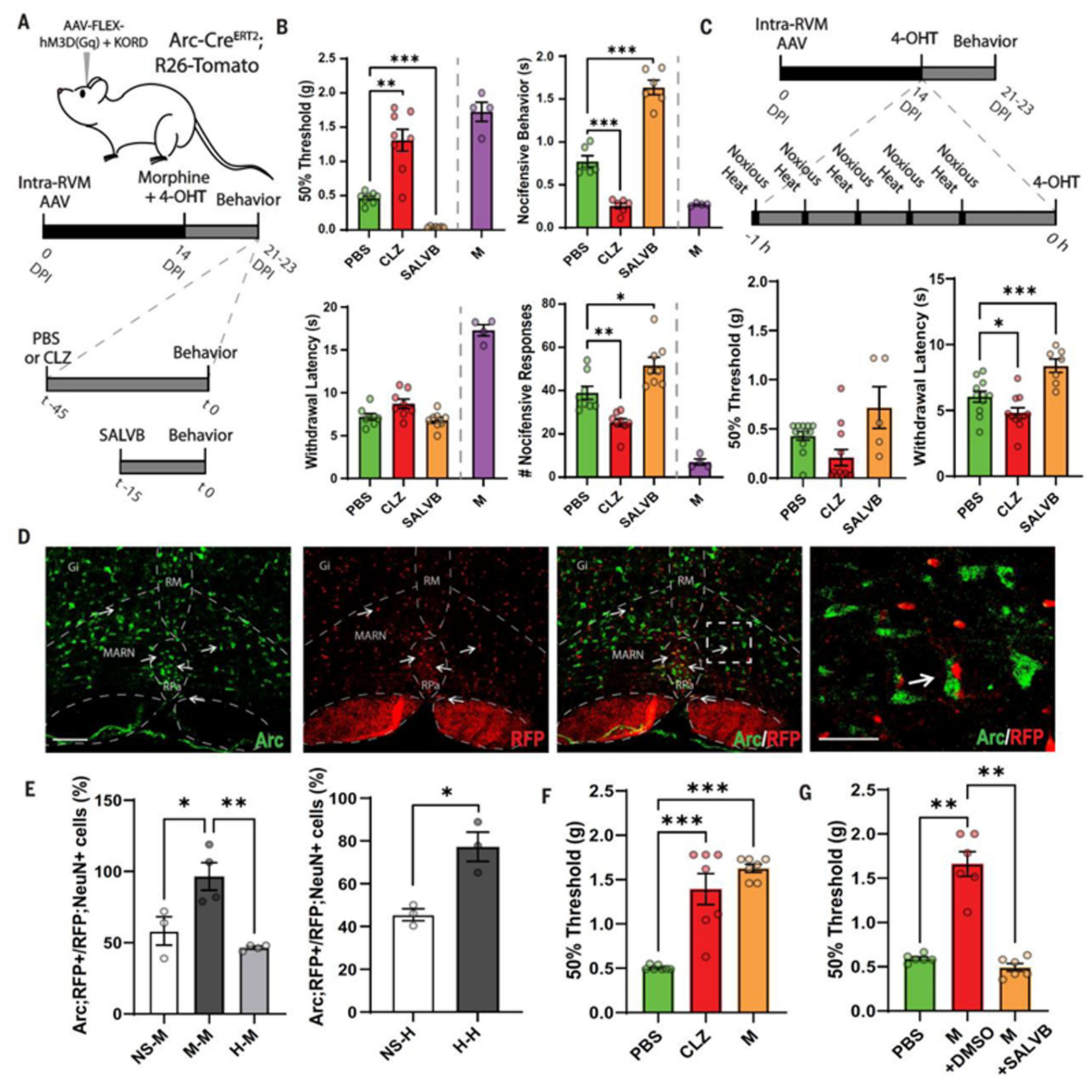
A morphine antinociceptive ensemble in the RVM. (**A**) Schematic outline of the method for TRAPing morphine-activated RVM neurons and synthetic activation to assess behavioral consequences. DPI, days postinjection; time (t) in minutes; 4-OHT, 4-hydroxy-tamoxifen. (**B**) Nociceptive thresholds and nocifensive behavior in mice administered PBS, or chemogenetic activation (CLZ) or inhibition (SALVB) of the morphine RVM ensemble as compared with morphine (M). (Top) von Frey withdrawal threshold and nocifensive behavior in response to noxious mechanical stimulation (1 g von Frey). *n* ≥ 6 mice per condition, **P < 0.01, ***P < 0.001. (Bottom) Heat withdrawal latency [Hargreaves 35% infrared (IR) intensity] and nocifensive responses to noxious thermal stimulation (hot plate) and effects of morphine. *n* = 8 mice per condition, *P < 0.05, **P < 0.01. (**C**) Schematic outline of the method for TRAPed heat active RVM neurons and synthetic activation to assess behavioral consequences. Time in hours. von Frey withdrawal threshold and heat withdrawal latency. (**D**) Coronal section through the RVM of an Arc-Cre^ERT2^;R26-Tomato mouse in which the analgesic ensemble had been captured (Arc protein, green; Tomato-RFP, red). Arrows indicate double-positivity. Dashed white box indicates higher magnification on the right. Gi, gigantocellular reticular nucleus; MARN, magnocellular reticular nucleus; RM, nucleus raphe magnus; RPa, nucleus raphe pallidus. Scale bar, 150 μm (40 μm for inset). (**E**) Quantification of double-positive neurons for Arc and Tomato (RFP) in Arc-Cre^ERT2^;R26-Tomato mice subjected to matched or mismatched stimuli. No stimulus (NS), morphine (M), or noxious heat stimuli (H). Arc;RFP and RFP;NeuN were quantified on adjacent sections. *n* ≥ 3 mice per condition, *P < 0.05, **P < 0.01. (**F**) Mechanical withdrawal threshold in mice administered PBS, CLZ, or morphine. *n* = 7 mice per condition, ***P < 0.001. (**G**) Mechanical withdrawal in mice administered PBS, morphine plus vehicle (M + DMSO), or morphine plus chemogenetic inhibition of the RVM analgesic ensemble (M + SALVB). *n* = 6 mice per condition, **P < 0.001. Error bars indicate SEM.

To access RVM neurons whose activity is altered by morphine we used stimulus-transcription coupling of immediate early gene Arc (Arc-Cre^ERT2^ TRAP;R26-Tomato mice) to permanently mark cell types (hereafter referred to as TRAPing). To synthetically increase and decrease the activity of morphine-responsive neurons in the same animal, we injected adeno-associated viruses (AAVs) carrying constructs for the Cre-dependent expression of the excitatory DREADD neuromodulator [hM3D(Gq), activated by clozapine (CLZ)] and the inhibitory κ-opioid DREADD [hKORD, activated by salvinorin B (SALVB)] (Fig. 1A and fig S1A). Synthetic stimulation of neurons that were TRAPed by morphine (10mg/kg subcutaneous, activation of “antinociceptive morphine RVM ensemble”) using CLZ (0.3mg/kg) increased the mechanical von Frey withdrawal threshold nearly threefold, and markedly reduced nocifensive behavior to noxious mechanical pricking with similar effect size as morphine itself (Fig. 1B, fig. S1B). Conversely, in the same animals, when these neurons were inhibited with SALVB, a marked allodynia and increased nocifensive behavior to noxious pricking was observed as compared with control mice, whereas activation and inhibition of a “control” ensemble TRAPed without stimulus or CLZ and SALVB administered to naïve wild-type (WT) mice had no effect (Fig. 1B and fig. S1, C and D). The morphine RVM ensemble attenuated heat-induced nocifensive behavior but had no effect on heat withdrawal latency (Fig. 1B). When noxious heat-activated RVM neurons were TRAPed and synthetically activated 1 week later, animals displayed allodynia and reduced withdrawal latency to radiant heat, and when these neurons were inhibited in the same animals, an increased withdrawal latency was observed (Fig. 1C and fig. S1, E and F). To validate the existence of antinociceptive and pronociceptive ensembles, neurons activated by morphine or noxious heat stimuli were TRAPed, 2 weeks later a second stimulus was administered, and animals were euthanized for quantification of neurons activated by the first stimulus (TRAPed neurons), the second stimulus [expression of the immediately early gene (IEG) *Arc* (activity regulated cytoskeletal-associated protein)], or both (Fig. 1D). No stimulus-dependent difference in the total number of active TRAPed neurons or Arc-positive neurons was observed in the Arc-Cre^ERT2^;R26-Tomato mice, suggesting increased and decreased expression among different populations (fig. S1, G and H). However, when morphine or heat neurons were first TRAPed, and animals were immediately euthanized after the second stimulus, an enrichment of Arc expression in TRAPed neurons was observed only in animals with the same stimulus delivered twice (Fig. 1E and fig. S1I). We then examined the relative contribution of the RVM to overall morphine-induced antinociception. Cre-dependent AAV hM3D(Gq) and hKORD were delivered to the RVM of Arc-Cre^ERT2^ mice, the analgesic RVM ensemble was captured with morphine, and 1 week later, animals received either morphine or CLZ. Activation of the morphine RVM ensemble resulted in an increase in mechanical threshold that was similar to the effect of morphine administered to the same animal (Fig. 1F). To identify whether the morphine RVM ensemble represents a critical anatomical substrate, we administered morphine and at the same time inhibited the RVM ensemble by SALVB. This completely abolished morphine-induced mechanical antinociception (Fig. 1G).

### Molecular identity of the antinociceptive RVM ensemble

**Fig 2:**
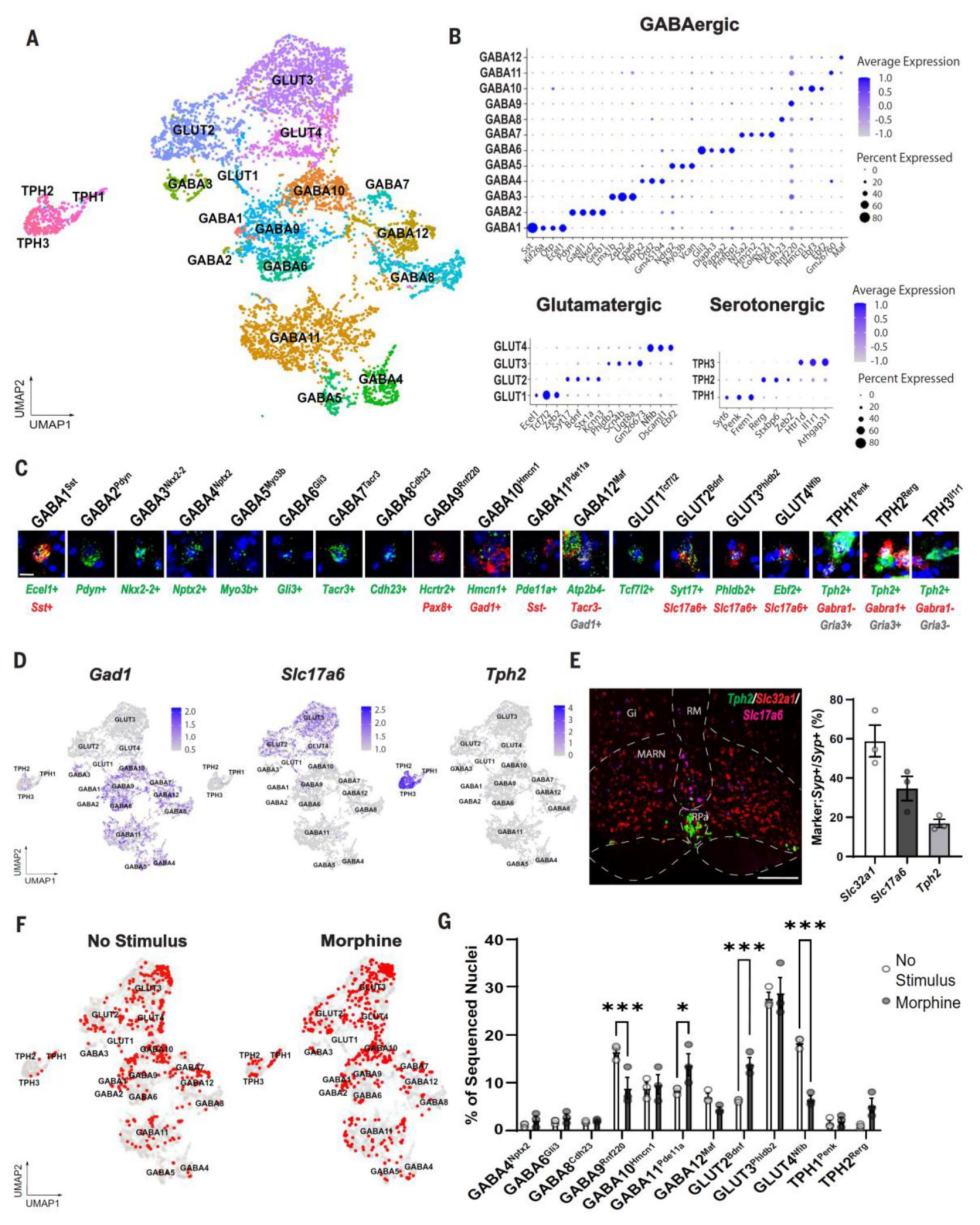
Molecular identity of the morphine antinociceptive RVM ensemble. (**A**) Uniform manifold approximation and projection (UMAP) plot of 6631 snRNA-seq clustered RVM neurons. (**B**) Marker expression in GABAergic, glutamatergic, or serotonergic neurons. (**C**) Validation of RVM neuron types using fluorescence in situ hybridization. Scale bar, 15 μm. (**D**) Feature plots of *Gad1, Slc17a6* (Vglut2), and *Tph2* expression in identified RVM neurons. (**E**) (Left) In situ hybridization of the RVM for *Slc32a1* (Vgat), *Slc17a6* (Vglut2), and *Tph2*. Abbreviations of nuclei as in Fig. 1D. Scale bar, 300 μm. (Right) Quantification of *Slc32a1*-, *Slc17a6*-, or *Tph2*-positive RVM cells as proportion of total *Syp*-positive neurons. *n* = 3 mice. (**F**) UMAP plot of nuclei isolated from RVM of Arc-Cre^ERT2^;R26-Tomato mice subjected to no stimulus (left) or morphine (right). Captured nuclei (red) were mapped onto the RVM atlas (gray). (**G**) Quantification of sequenced nuclei. *n* = 3 samples per condition, and at least five mice were used per sample. *P < 0.05, ***P < 0.001. Error bars indicate SEM.

We performed single-nucleus RNA sequencing (snRNA-seq) to obtain information on the neuronal types representing the morphine RVM ensemble. A reference taxonomy of RVM neurons was first established (Fig. 2A and fig. S2). A total of 6631 sequenced nuclei with an average of 4030 genes and 12,992 transcripts detected per nucleus were included. Clustering revealed 4 distinct glutamatergic, 3 serotonergic, and 12 GABAergic neuron types (Fig. 2B), all validated as RVM neuronal types by RNAscope in situ hybridization (Fig. 2C and figs. S3 and S4). *Slc17a6* (Vglut2), *Tph2*, and *Gad1* were expressed in the predicted clusters in a near mutually exclusive manner (~20% of *Tph2*-expressing neurons also expressed *Slc17a6*), confirming neurons with glutamatergic, serotonergic, and GABAergic neurotransmission, respectively (Fig. 2D). Quantification of the proportion of the different neurotransmitter-expressing neurons in the RVM by in situ hybridization showed inhibitory (*Slc32a1*-positive) neurons to be the most abundant, glutamatergic (*Slc17a6*-positive) neurons as intermediate, and serotonergic (*Tph2*-positive) neurons as the least frequent type in the RVM, consistent with the RNA-seq data (Fig. 2E). Among RVM neurons, *Oprm1* was expressed in 8 of the 12 GABAergic neuron types, two of the three serotonergic types, and all glutamatergic types (fig. S5A). We next TRAPed and prospectively isolated RVM neurons from Arc-Cre^ERT2^;R26-Tomato mice for snRNA-seq of positive nuclei from control mice (“No Stimulus”) and mice that had received morphine. The cell type identity of the sequenced neurons was obtained using label transfer from our reference atlas (Fig. 2F; see Materials and methods). Most neuronal types were not TRAPed in control mice. However, some neuronal types, including GABA9 to GABA12 and GLUT2 to GLUT4, represented a significant proportion in the control mice, indicating activity in the naïve mouse (Fig. 2G). In morphine-TRAPed animals, most neuronal types were weakly represented, as in control mice, and were unaffected by morphine, with the exception of a few TPH2 neurons. Instead, the morphine antinociceptive ensemble was represented by a change in TRAPed neurons among four neuron types also TRAPed in control mice. An increase in TRAPed neurons was seen in one inhibitory (GABA11) and one excitatory (GLUT2) neuron type, and a decrease was seen in one inhibitory (GABA9) and one excitatory (GLUT4) neuron type (Fig. 2G).

### Morphine antinociception elicited by activation of excitatory RVM neurons projecting to the spinal cord

**Fig 3:**
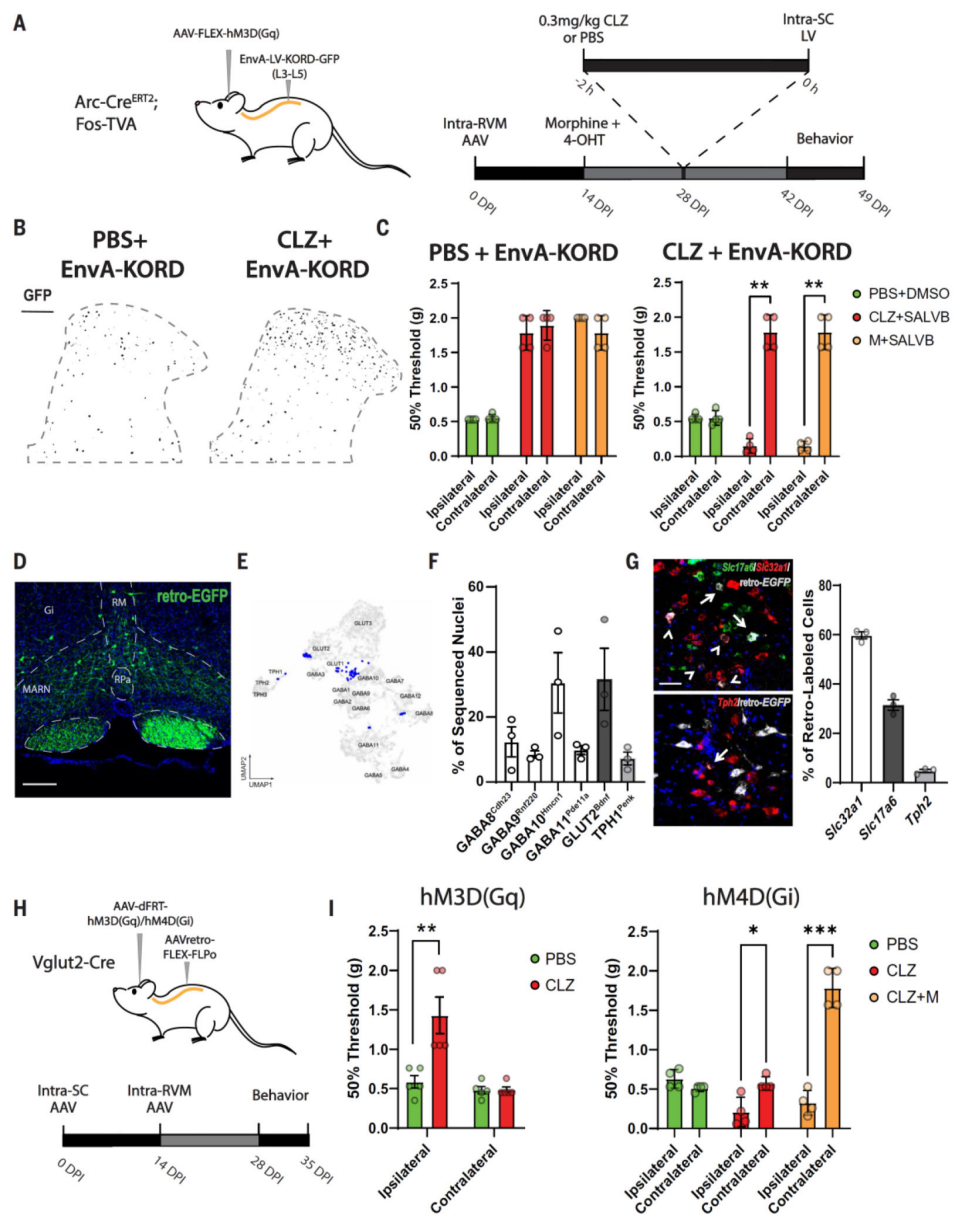
Morphine antinociception elicited by activation of excitatory RVM neurons projecting to the spinal cord. (**A**) Schematic outline for unilateral TRAP of active spinal neurons in response to chemogenetic activation of the RVM analgesic ensemble and behavioral protocol. LV, lentivirus; SC, spinal cord. (**B**) Coronal sections through the dorsal horn of the lumbar spinal cord showing captured neurons (GFP, black) in response to either PBS or CLZ. Gray dashed line indicates spinal gray matter. Scale bar, 100 μm. (**C**) Mechanical withdrawal response in Arc-Cre^ERT2^;Fos-TVA mice in which spinal neurons were captured in response to either vehicle (left, PBS + EnvA-KORD) or activation of the RVM analgesic ensemble (right, CLZ + EnvA-KORD). Measurements in mice during baseline conditions (PBS + DMSO), activation of the RVM analgesic ensemble and inhibition of captured spinal neurons (CLZ + SALVB), or morphine and inhibition of captured spinal neurons (M + SALVB). *n* = 4 mice per condition, **P < 0.001. (**D**) Image from the RVM of a mouse with unilateral AAVretro-enhanced green fluorescent protein (EGFP) injection into the lumbar spinal cord. EGFP-positive (green) are spinal projection neurons. Abbreviations of nuclei as in Fig. 1D. Scale bar, 200 μm. (**E**) UMAP plot showing EGFP-positive cells isolated and RNA sequenced (blue; reference atlas in gray) from the RVM of mice injected intraspinal with AAVretro-EGFP. (**F**) Quantification of sequenced cells. *n* = 3 separate samples, at least three mice per sample. (**G**) In situ hybridization of the RVM from mice with unilateral AAVretro-EGFP injection into the lumbar spinal cord. (Left) *Slc17a6* (top, green), *Slc32a1* (top, red), or *Tph2* (bottom, red) together with retro-*EGFP* (gray) in situ hybridization. Arrows indicate retro*-EGFP* double-positive cells with *Slc17a6* or *Tph2*. Arrowheads indicate cells double-positive for retro*-EGFP* and *Slc32a1*. Scale bar, 50 μm. (Right) Quantification. *n* = 3 mice. (**H**) Schematic outline for unilateral capture of spinally projecting Vglut2-positive neurons in the RVM. (**I**) (Left) Mechanical withdrawal threshold in mice with PBS or chemogenetic activation of spinally projecting Vglut2-positive cells (CLZ). (Right) Mechanical withdrawal threshold in mice with PBS, inhibition (CLZ), or inhibition of spinally projecting Vglut2-positive cells and morphine (CLZ+M). *n* = 4 or 5 mice per condition, *P < 0.05, **P < 0.01, *** *P* < 0.001. Error bars indicate SEM.

Some of the RVM neurons are expected to project to the spinal cord, where they gate the incoming ascending sensory system, thereby modulating nociception (5). To directly address whether descending projection neurons confer mechanical antinociception by engaging spinal interneurons, we established a mouse model where we could TRAP morphine-activated RVM neurons and synthetically reactivate these neurons while simultaneously capturing spinal neurons activated specifically by the RVM ensemble. We combined the TRAP method with the CANE (capturing activated neuronal ensembles) technology (17), enabling dual activity–dependent capture of neuronal ensembles. CANE is based on a destabilized TVA virus receptor expressed from the IEG Fos. An EnvA-pseudotyped lentivirus introduced into the spinal cord within a short time window that parallels the expression of the destabilized TVA ensures that only very recently excited neurons become infected. Arc-Cre^ERT2^ mice crossed with Fos-TVA mice were injected with AAV carrying a Cre-dependent hM3D(Gq) into the RVM, and hM3D(Gq) expression was TRAPed in neurons active during morphine administration. To capture RVM-responsive spinal neurons, an EnvA-pseudotyped lentivirus expressing KORD and green fluorescent protein (GFP) was injected into the spinal cord, while also administering either vehicle or CLZ to activate the RVM morphine ensemble (Fig. 3A). Activation of the morphine RVM ensemble during spinal delivery (CLZ+EnvA-KORD) led to a marked increase in GFP-expressing neurons in ipsilateral superficial laminae of the dorsal horn, as compared with control mice (PBS+EnvA-KORD), where spinal neurons were captured without administration of CLZ (Fig. 3B). While neurons were TRAPed bilaterally in the RVM, the spinal EnvA-pseudotyped lentivirus was injected unilaterally, thus the contralateral side of the animal served as an additional control. To assess the functional role of these spinal neurons in morphine antinociception, we tested mechanical thresholds under control conditions [phosphate-buffered saline (PBS)+dimethyl sulfoxide (DMSO)] or after inhibition of this TRAPed spinal ensemble in combination with activation of the morphine RVM ensemble (CLZ+SALVB) or morphine (M+SALVB). In control mice, where spinal neurons were captured without activation of the RVM ensemble, inhibition of the spinal ensemble did not diminish the mechanical antinociception of either activation of the RVM ensemble (CLZ) or morphine (M) administration. However, inhibition of spinal neurons TRAPed after activation of the morphine RVM ensemble completely abolished the effect of both activation of the RVM ensemble (CLZ) and morphine on mechanical withdrawal ipsilateral, but not contralateral, to the spinal injection (Fig. 3C). Furthermore, the animals displayed increased sensitivity (allodynia), suggesting that the RVM controls spinal neurons that continuously modulate nociception in the ascending pathway. To determine the molecular identities of those neuron types, which project from the RVM to the spinal cord, we injected AAVretro-EGFP (enhanced green fluorescent protein) into the lumbar spinal cord, which led to robust labeling of RVM neurons (Fig. 3D). Prospective isolation of the EGFP-expressing neurons and single-cell RNA sequencing (scRNA-seq), followed by label transfer to the reference atlas, revealed four inhibitory, one glutamatergic, and one serotonergic RVM projection neuron type (Fig. 3, E and F). These included the two inhibitory neuron types displaying morphine-induced changes, whereby morphine led to decreased GABA9 and increased GABA11 TRAPed neurons (Fig. 2G). Beyond these, the only other RVM projection neuron type converging with morphine-induced alteration was GLUT2. The proportion of GABAergic, glutamatergic, and serotonergic projection neurons was quantified in retrogradely traced mice by in situ hybridization for *Slc32a1, Slc17a6*, and *Tph2*, respectively. Around 60, 32, and 5% of RVM projection neurons were GABAergic, glutamatergic, and serotonergic neurons, respectively (Fig. 3G), consistent with the scRNA-seq data (fig. S5B). Nonetheless, most of the GABAergic projection neurons were GABA10, which were morphine unresponsive. Thus, we conclude that glutamatergic RVM GLUT2 neurons represent a considerable proportion of the morphine-directed output from the RVM. We therefore examined the behavioral consequences of activating and inhibiting specifically the excitatory RVM projection neurons using intersectional genetics. An AAVretro-FLEX-FLPo virus was administered to the lumbar spinal cord, and an AAV with FLP-dependent hM3D(Gq) or hM4D(Gi) to the RVM, of Vglut2-Cre mice (Fig. 3H). CLZ activation increased the mechanical threshold ipsilateral, but not contralateral, to the spinal injection, whereas inhibition led to allodynia. Administration of morphine while simultaneously inhibiting the excitatory projection neurons abolished morphine’s antinociceptive effect (Fig. 3I). The effect size on mechanical antinociception was similar to that observed when manipulating activity of the morphine antinociceptive RVM ensemble.

### BDNF/TrkB-dependent morphine antinociception by RVM^BDNF^ neurons

**Fig 4:**
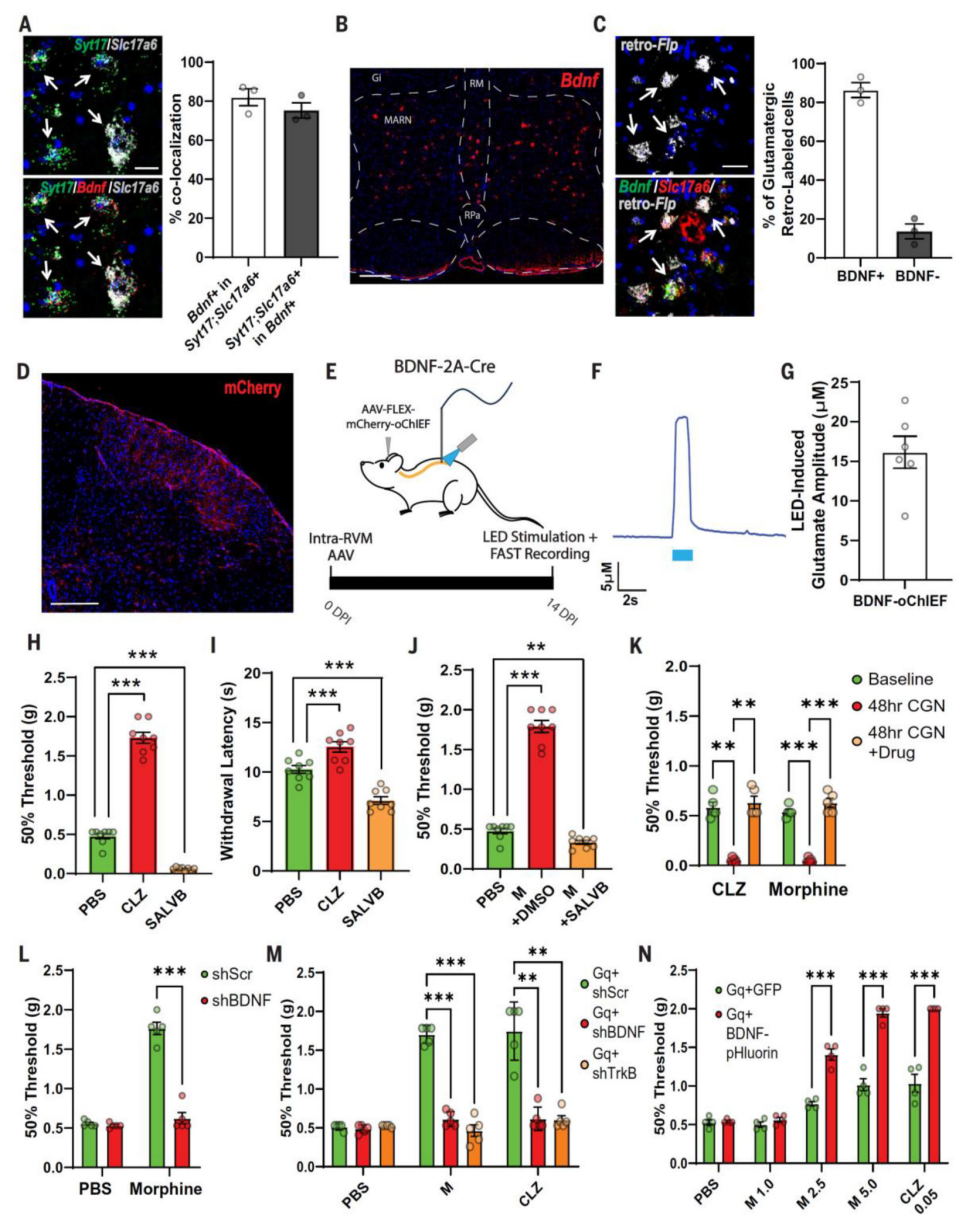
BDNF/TrkB-dependent morphine antinociception by RVM^BDNF^ neurons. (**A**) In situ hybridization of the RVM of WT mice. (Left) Neurons coexpressing *Syt17* (green), *Slc17a6* (gray), and *Bdnf* (bottom, red). Arrows indicate triple-positive cells. Scale bar, 20 μm. (Right) Quantification of *Bdnf*;*Syt17*;*Slc17a6*-positive cells. *n* = 3 mice. (**B**) In situ hybridization for *Bdnf* in the RVM. Abbreviations of nuclei as in Fig. 1D. (**C**) In situ hybridization of the RVM in mice with spinal AAVretro-Flp injection. (Left) Neurons coexpressing retro*-Flp* (gray), *Bdnf* (green), and *Slc17a6* (red). Arrows indicate triple-positive cells. Scale bar, 30 μm. (Right) Quantification of *Bdnf-positive* and *Bdnf*-*negative*;retro*-Flp-* and *Slc17a6-positive* cells as indicated. *n* = 3 mice. (**D**) RVM^BDNF^ axon projections in superficial laminae of the cervical spinal cord (mCherry). Scale bar, 200 μm. (**E**) Schematic outline for (F) and (G). (**F**) Trace of optogenetically induced glutamate amplitude in the lumbar spinal. (**G**) Quantification of glutamate amplitude in the lumbar spinal. *n* = 6 mice. (**H**) Mechanical and (**I**) thermal (Hargreave’s 30% IR intensity) withdrawal thresholds in mice administered PBS or CLZ activation or SALVB inactivation of RVM^BDNF^ neurons. *n* = 8 mice per condition, ***P < 0.001. (**J**) Mechanical withdrawal thresholds in mice administered PBS, morphine plus vehicle (M + DMSO), or morphine plus inhibition of RVM^BDNF^ neurons (M + SALVB). *n* = 6 mice per condition, ***P < 0.001. (**K**) Mechanical threshold in mice with CLZ inactivation of RVM^BDNF^ neurons (CLZ) or morphine in mice 48 hours after carrageenan (CGN, intraplantar injection). (**L**) Mechanical withdrawal threshold after PBS or morphine (M) in mice with AAV for scrambled shRNA (shScr) or shRNA against BDNF (shBDNF) in the RVM. *n* = 5 mice per condition, ***P < 0.001. (**M**) Mechanical withdrawal threshold in mice with PBS, morphine (M), or activation of RVM^BDNF^ neurons (CLZ) in BDNF-2A-Cre mice which have Cre-dependent expression of hM3D(Gq) and either scrambled shRNA (Gq + shScr), shRNA for BDNF (Gq + shBDNF), or shRNA for TrkB (Gq + shTrkB) in the RVM. *n* = 5 mice per condition, **P < 0.01, ***P < 0.001. (**N**) Mechanical withdrawal threshold in mice administered PBS or morphine (M 1.0 = 1.0 mg/kg, M 2.5 = 2.5 mg/kg, M 5.0 = 5.0 mg/kg) or subthreshold activation of RVM^BDNF^ neurons (CLZ 0.05 = 0.05 mg/kg clozapine) in BDNF-2A-Cre mice with Cre-dependent expression of hM3D(Gq) and either GFP (Gq + GFP), or pHluorin-tagged BDNF (Gq + BDNF-pHluorin). *n* = 4 mice per condition, ***P < 0.001. Error bars indicate SEM.

The finding that excitatory RVM projection neurons substantially contribute to morphine antinociception, and that the GLUT2 neuron type is the only excitatory projection neuron, suggested that this neuronal type is essential for mechanical antinociception elicited by morphine. Brain-derived neurotrophic factor (BDNF) was enriched in these neurons (Fig. 2B). Triple in situ hybridization for *Syt17* and *Slc17a6*, a combination uniquely expressed in GLUT2 neurons, and *Bdnf* revealed that most *Bdnf*-expressing neurons expressed *Syt17* and *Slc17a6* and, conversely, most *Syt17-* and *Slc17a6-*expressing neurons expressed *Bdnf* (Fig. 4A). Thus, GLUT2 neurons are represented by RVM^BDNF^ neurons distributed along the rostrocaudal extent of the RVM (Fig. 4B and fig. S6). RVM^BDNF^ neurons were confirmed to represent the major glutamatergic projection neuron type in the RVM using AAVretro-FLPo injected into the spinal cord and quantifying *FLPo* colocalization with *Bdnf* and *Slc17a6*. More than 80% of all retrogradely labeled excitatory *Slc17a6*-positive cells were *Bdnf*-positive (Fig. 4C). To specifically examine the role of RVM^BDNF^ neurons on morphine antinociception, we used the previously characterized BDNF-2A-Cre knock-in mouse line (18), wherein Cre recombinase expression mimics endogenous BDNF expression. To visualize the spinal projections of RVM^BDNF^ neurons, we injected an AAV expressing Cre-dependent mCherry into the RVM of these mice. A strong innervation pattern throughout spinal cord laminae I and II was observed (Fig. 4D), where ascending nociceptors terminate for processing and relay to the parabrachial nucleus and thalamus. To establish functional glutamatergic neurotransmission by RVM^BDNF^ neurons, a Cre-dependent ChR2 variant (oChIEF) was expressed in these neurons, and activity was controlled in spinal terminals using optogenetics. Depolarization-evoked extracellular glutamate levels were measured using the fast analytical sensing technology (FAST) in vivo in the anesthetized animals (19). By applying a light pulse to the surface of the spinal cord, we observed a rapid increase in glutamate release, with a peak amplitude of ~16 μM (Fig. 4, E to G). Significantly weaker glutamate recordings were observed when the light intensity was reduced, thus confirming that the observed glutamate release is due to the activation of oChIEF-expressing axon terminals (fig. S7, A and B).

To determine the effects of RVM^BDNF^ neurons on nociception, we administered an AAV carrying Cre-dependent expression of hM3D(Gq) and hKORD in the RVM of BDNF-2A-Cre knock-in mice. Activating and inhibiting RVM^BDNF^ neurons led to a significant increase and decrease, respectively, in the proportion of Fos-positive RVM^BDNF^ neurons (fig. S7C). Activation of RVM^BDNF^ neurons led to increased mechanical threshold (Fig. 4H) similar to what was observed for activation of the morphine-captured antinociceptive ensemble. Conversely, in the same animals, inactivation of RVM^BDNF^ neurons using SALVB led to a marked allodynia to mechanical stimuli (Fig. 4H), mimicking inactivation of the entire morphine ensemble (Fig. 1B) and suggesting an ongoing suppression of nociception in naïve mice. Activation and inhibition of RVM^BDNF^ neurons, unlike the morphine ensemble, significantly affected the heat withdrawal latency (Fig. 4I). To establish whether RVM^BDNF^ neurons are central, we administered vehicle (DMSO) or SALVB and quantified the antinociceptive effect of morphine on mechanical sensitivity. While morphine led to a pronounced elevated threshold in control mice, inhibition of RVM^BDNF^ neurons completely prevented mechanical antinociception by morphine (Fig. 4J). However, although morphine antinociception was completely prevented, there was a difference in outcome as compared with inhibition of RVM^BDNF^ neurons without morphine, as those mice also displayed allodynia (Fig. 4H). This discrepancy is likely due to a residual contribution of other, non-RVM^BDNF^ neurons to mechanical antinociception by morphine. Inflammation-induced allodynia produced by carrageenan (CGN) was reversed by both morphine and activation of RVM^BDNF^ neurons (Fig. 4K and fig. S7D), suggesting an importance of RVM^BDNF^ neurons also during situations of sensitization. BDNF plays a crucial role in synaptic transmission (20–22). The conspicuous expression of BDNF only in RVM^BDNF^ neurons led us to examine its role in morphine antinociception. Mice treated with intra-RVM short hairpin RNA (shRNA) for BDNF revealed that, 2 weeks after injection, morphine had no antinociceptive effect on mechanical thresholds, while baseline mechanical nociceptive sensitivity remained unchanged. Conversely, morphine remained efficacious in mice receiving scrambled shRNA (Fig. 4L). To determine whether BDNF is essential within the antinociceptive RVM ensemble and to resolve whether it acts trans-synaptically or in an autocrine fashion (20), we injected Cre-dependent AAV hM3D(Gq) into the RVM of BDNF-2A-Cre mice and at the same time also administered an AAV carrying Cre-dependent shRNA for either BDNF, its cognate receptor TrkB [NTRK2 (neurotrophic receptor tyrosine kinase 2)], or a scrambled control. While morphine had the expected antinociceptive effect on mechanical threshold in scrambled shRNA control mice, it had no effect in BDNF or TrkB shRNA mice when tested 2 weeks later (Fig. 4M). Similar results were obtained when we selectively activated RVM^BDNF^ neurons using CLZ (Fig. 4M). To address whether BDNF can potentiate morphine antinociception, we injected BDNF-2A-Cre mice with a Cre-dependent hM3D(Gq) and either an AAV encoding Cre-dependent BDNF overexpression (Gq + BDNF-pHluorin) or GFP control (Gq + GFP). BDNF overexpression in the RVM^BDNF^ neurons markedly potentiated mechanical antinociception by morphine, with efficacy at doses where morphine alone was insufficient (Fig. 4N). Furthermore, when RVM^BDNF^ neurons were activated using low doses of CLZ (0.05 mg/kg) with minimal effects by itself, full efficacy was observed in animals with BDNF overexpression in RVM^BDNF^ neurons (Fig. 4N).

### Spinal mechanisms of morphine antinociception

**Fig 5:**
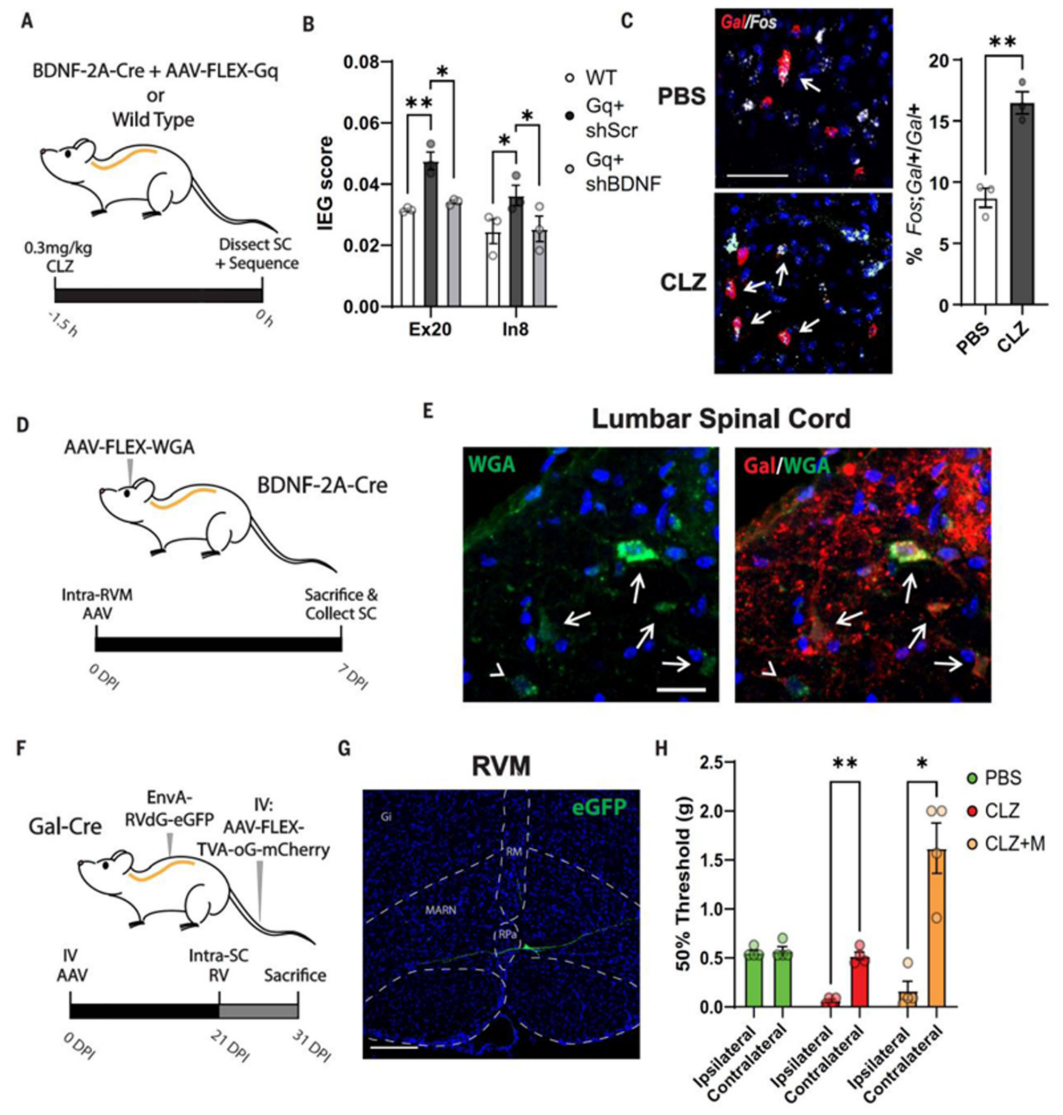
Spinal mechanisms of morphine antinociception. (**A**) Schematic outline for TRAPing and snRNA-seq of spinal cord neurons after activation of RVM^BDNF^ neurons, analyzed in (**B**). (B) IEG module score of 103 IEGs in mice without (WT) or with activation of RVM^BDNF^ neurons and scrambled shRNA (Gq + shScr) or activation of RVM^BDNF^ neurons and shRNA knockdown of BDNF expression (Gq + shBDNF). *n* = 3 mice per condition, *P < 0.05, **P < 0.01. (**C**) In situ hybridization of *Fos* (gray) and galanin (*Gal*, red) in lumbar spinal cord of mice, as in (A), administered PBS or CLZ. (Left) Images. (Right) Quantification. Arrows denote double-positive cells. Scale bar, 50 μm. *n* = 3 mice per condition, **P < 0.01. (**D**) Schematic outline of anterograde tracing. (**E**) Immunofluorescence images of a lumbar spinal cord section stained for wheat germ agglutinin (WGA, green) and galanin (red) showing anterograde tracing from RVM^BDNF^ neurons. Arrows indicate double-positive cells; arrowheads, WGA-positive, Gal-negative cells. Scale bar, 20 μm. (**F**) Schematic outline of monosynaptic retrograde tracing from Gal-positive spinal cord cells. *EnvA-RvdG*: envelope A pseudotyped, G-deleted rabies virus, *TVA-oG-mCherry*: TVA-optimized G-mCherry. (**G**) EnvA-pseudotyped rabies virus expressing EGFP in the RVM traced from galanin-positive lumbar spinal cord cells. Abbreviations of nuclei as in Fig. 1D. Scale bar, 20 μm. (**H**) Mechanical withdrawal threshold of Gal-Cre mice with spinal Cre-dependent hM4D(Gi) expression administered either PBS, CLZ, or CLZ and morphine (CLZ+M). *n* = 4 mice per condition, *P < 0.05, **P < 0.001. Error bars indicate SEM.

The finding that the RVM elicits antinociception through spinal projection neurons indicates the engagement of specific spinal neuron types processing antinociception. To identify the neural basis for antinociception elicited by spinal neurons, we took either WT mice or BDNF-2A-Cre mice injected with a Cre-dependent AAV-hM3D(Gq) in the RVM and 2 weeks later administered CLZ (Fig. 5A). After CLZ treatment, we dissected the spinal cord in the presence of the transcription blocker actinomycin D and performed snRNA-seq. A collection of 103 known IEGs (23) was used to establish integrated module and enrichment scores (see Materials and methods) within dorsal horn neuron types as an indicator of neural activity. For cell type probabilistic analysis and cell type annotation propagation, we used our recent high-coverage scRNA-seq reference spinal cord atlas (24). We compared the module score of WT mice receiving CLZ with mice receiving CLZ activation of RVM^BDNF^ neurons, as well as IEG positivity and enrichment relative to WT mice. The only neuronal types with increased scores in all analyses were Excitatory 20 (Ex20) and Inhibitory 8 (In8), which had altered IEG module scores (Fig. 5B) and increased enrichment and IEG positivity as measures of activity (fig. S8, A to D). To examine the role of BDNF, we injected shRNA BDNF along with the Cre-dependent AAV-hM3D(Gq) in the RVM of BDNF-2A-Cre mice. CLZ activation of RVM^BDNF^ neurons while silencing BDNF reversed the increase in IEG module score and IEG positivity (Fig. 5B and fig. S8E). Because the antinociceptive RVM neurons were excitatory, we focused on inhibitory spinal neurons that could explain the antinociception elicited by RVM^BDNF^ neurons. In the single-cell analysis of spinal neurons, In8 neurons were the only neuron type expressing galanin (SC^Gal^ neurons). To validate whether RVM^BDNF^ neurons can activate SC^Gal^ neurons in vivo, we used in situ hybridization to examine whether activation of RVM^BDNF^ neurons leads to an increased number of SC^Gal^ neurons expressing the IEG *Fos*. We compared *Fos*-expressing SC^Gal^ neurons in BDNF-2A-Cre mice with intra-RVM injection of a Cre-dependent AAV-hM3D(Gq) followed by vehicle or CLZ administration. The activation of RVM^BDNF^ neurons led to a significant increase in the proportion of *Fos*-positive SC^Gal^ neurons (Fig. 5C). To determine whether RVM^BDNF^ cells directly contact SC^Gal^ neurons, an AAV encoding Cre-dependent anterograde tracer wheat germ agglutinin (WGA) was injected into the RVM of BDNF-2A-Cre mice (Fig. 5D). Analysis of the lumbar spinal cord 7 days later showed many neurons positive for both galanin and WGA (Fig. 5E), indicating that RVM^BDNF^ neurons synapse onto SC^Gal^ neurons. To further determine whether SC^Gal^ neurons have direct connectivity with RVM neurons, we injected mice with Cre recombinase expression in galanin-expressing cells (Gal-Cre mice) with an AAV carrying Cre-dependent TVA-oG, thus allowing for expression of TVA-oG in SC^Gal^ neurons (see Materials and methods). Specifically, we used the PHP.eB serotype for intravenous injection, which has tropism for central rather than peripheral nervous system neurons (25). Three weeks later, the mice received an intraspinal injection of an EnvA-pseudotyped, glycoprotein (G)–deleted rabies virus expressing EGFP for monosynaptic tracing of input neurons to SC^Gal^ neurons (Fig. 5F). Neurons with monosynaptic input to SC^Gal^ neurons (EGFP-positive) were observed primarily in the RVM (three of three mice), but several cells were also located in the locus coeruleus (one of three mice; Fig. 5G and fig. S8F). Finally, we examined whether SC^Gal^ neurons play a role in morphine-based mechanical antinociception. Gal-Cre mice were injected in the lumbar spinal cord with a Cre-dependent AAV expressing hM4D(Gi). Two weeks later, the mice were injected with either vehicle (PBS), clozapine to inhibit SC^Gal^ neurons (CLZ), or clozapine together with morphine (CLZ+M), and mechanical sensitivity was assessed. Notably, whereas CLZ resulted in marked allodynia on the ipsilateral side, morphine antinociception was completely abolished by chemogenetic inhibition of SC^Gal^ cells (Fig. 5H). Thus, we conclude that GABAergic SC^Gal^ neurons receive monosynaptic inputs and are recruited by RVM^BDNF^ projection neurons and that morphine antinociception is elicited by recruitment of GABAergic SC^Gal^ neurons, which inhibit spinal processing of mechanical nociception.

## Discussion

Our study examined the mechanism underlying morphine antinociception by using several recent technological advances, including the dual activity–dependent approach ArcTRAP and CANE, snRNA-seq, computational analyses, monosynaptic tracing, and behavioral assays of neuronal activity. We implicate a small neural ensemble in the RVM as central for mechanical antinociception by morphine, and that also affects heat-induced nociception. Neural activity alone in the RVM induced the key features of morphine-induced mechanical antinociception, and when inhibited, morphine had little effects. Molecularly defined glutamatergic RVM^BDNF^ projection neurons within the RVM ensemble engaged spinal inhibitory Gal-positive neurons and conferred antinociception.

μ-Opioid receptors are present in virtually all neural substrates of the brain and brainstem pain pathway that has direct or indirect connectivity with the RVM (1). Thus, μ-receptor engagement by morphine may include neurons within but also beyond the RVM, and consequently alter activity of antinociceptive OFF cells and pronociceptive ON cells that may contribute to antinociception (8). OFF cells pause and ON cells burst in association with nocifensive withdrawal, thus OFF cells are silent when ON cells are active and vice versa (26–28). Silencing the entire RVM produces hyperalgesia, indicating that this effect is due to a loss of OFF cell output (29). Morphine and μ-opioid receptor agonists lead to sustained activation of OFF cells and suppression of ON cells (28–30). The direct relation between ON and OFF cells as defined by electrophysiological recordings and TRAPed neurons (TRAPed ON and OFF cells, abbreviated tON and tOFF cells, respectively) is unknown. However, a direct relation between increased rates of activity and cessation of firing with increased and decreased immediate early gene expression has previously been observed in neurons with burst-like activity, such as RVM ON and OFF cells (31). Thus, we assumed an increased and decreased probability of TRAPed tOFF and tON neurons, respectively, by morphine. We found that GABA9 and GLUT4 were tON neurons, whereas GABA11 and GLUT2 were tOFF neurons. Among these, GABA9, GABA11, and GLUT2 were spinal projection neurons, suggesting parallel inhibitory and excitatory descending processes triggered by morphine, rather than representing an intra-RVM circuit. The finding of tON and tOFF GABAergic neurons is consistent with previous identification of the existence of GABAergic ON and OFF cells (32) and could explain conflicting results regarding pronociception versus antinociception elicited by GABAergic RVM neurons (10, 33, 34). Finally, we found that the GLUT4 tON cells were not spinal projection neurons and thus were local interneurons or projection neurons to regions other than the spinal cord.

The fluctuations in ON and OFF cell activity are believed to confer quantitative alterations in nociceptive sensitivity (35). On the basis of TRAPed neurons, we did not find the recruitment of any silent neuron type in the RVM by morphine. Instead, the RVM ensemble was represented by continuously active parallel pathways, and small shifts in the balance of GABA9 and GLUT4 tON cells and GABA11 and GLUT2 tOFF cells could modulate nociception. The identification of the RVM pronociceptive and antinociceptive neurons opens the way for exploration of not only the interactions within the RVM itself but also whether the identified parallel pathways are recruited during chronic pain and by cognitive, affective, and motivational components that modulate pain—areas that are almost entirely unexplored.

The RVM^BDNF^ neurons described here explain the mechanical antinociceptive effects of morphine. The results highlight the importance of excitatory neurotransmission by RVM^BDNF^ neurons and suggest minor, if any, roles for inhibitory tON and tOFF RVM projection neurons (GABA9 and GABA11, respectively). Furthermore, all GABAergic neurons affected by morphine were projection neurons. This suggests that tON cells have no effect on inactivation of RVM^BDNF^ neurons (which are tOFF cells), as has been previously suggested for ON and OFF cells (36). The powerful mechanical antinociception and pronounced pronociception elicited by activation and inhibition, respectively, of RVM^BDNF^ neurons suggest a continuous suppression of nociception in the naïve animal and the importance of inactivation for protective reflex and pain behavior to occur. Quantitative aspects of RVM^BDNF^ tOFF cell pause may determine the outcome, and in this way represent a continuous scaling of mechanical nociception through the descending pathway. This contrasts with morphine, which produces antinociception through forced activity of the RVM^BDNF^ tOFF cells. Individual RVM neurons can innervate the ipsilateral or contralateral spinal cord, such that the RVM at the population level displays diffuse widespread bilateral innervation (37, 38). Furthermore, some individual GABAergic and serotonergic neurons contain bilateral collaterals, whereas others do not (39, 40). Our behavioral data are consistent with a mostly ipsilateral spinal termination field of the glutamatergic RVM^BDNF^ neurons.

Inhibition of spinal neurons previously activated by RVM^BDNF^ neurons abolished both antinociception by activation of RVM^BDNF^ neurons and by morphine. This places the executive function of morphine antinociception at the excitatory connection of RVM^BDNF^ neurons with inhibitory spinal neurons, rather than presynaptic inhibition of primary afferents, consistent with the finding that RVM inputs onto primary afferents are either serotonergic or GABA/glycinergic but not glutamatergic (12). The pathway involves the monosynaptic recruitment of GABAergic spinal Gal-positive neurons by RVM^BDNF^ neurons. The importance of inhibition at the level of the spinal cord is well studied as a mechanism operating in the ascending pathway, where it maintains appropriate activity levels within spinal neuronal ensembles by regulating primary sensory input en route to ascending and reflex output pathways. This regulation is achieved through a convergence of feedforward inhibition and the interdependence of the spinal network components, which together shape the spinal output signal and nociceptive threshold to noxious stimuli (41). Recently, spinal cord neurons were molecularly classified using scRNA-seq (24, 42, 43). Gal-positive In8 neurons are located superficially in laminae I and II of the dorsal horn (24, 42). Gal-containing interneurons up-regulate phosphorylated extracellular signal-regulated kinases (pERK) and show altered expression of activity-regulated genes after noxious mechanical, thermal or chemical stimuli (42, 44). In this way, the In8 Gal-positive neurons identified in our study seem to link directly to a general inhibitory mechanism of nociceptive processing in the ascending pathway, irrespective of modality (24).

While all RVM neurons express TrkB (Ntrk2) in our snRNA-seq dataset, the critical role of BDNF and TrkB in the morphine antinociceptive ensemble was nevertheless unexpected. BDNF release and TrkB activation can take place in the pre- or postsynapse, where it potentiates glutamatergic transmission. Attenuation of post- but not presynaptic BDNF signaling is expected to affect activity of RVM output neurons. In such a case, forced activation of RVM^BDNF^ neurons that occurs independent of a putative postsynaptic action should overcome an experimental depletion of BDNF/TrkB and effectively elicit antinociception. We found a failure of forced activation of RVM^BDNF^ neurons to elicit any antinociception in mice with BDNF and TrkB silenced. This indicates a presynaptic function in the spinal cord. Our results underscore the divergence in the effects of TrkB^+^ neurons in the RVM, as likely a distinct subset receive input from BDNF-expressing neurons of the periaqueductal gray that exerts a postsynaptic pronociceptive effect contributing to inflammatory-induced pain hypersensitivity (45). At the presynaptic location, BDNF increases the probability of glutamate release (22, 46, 47) in a TrkB signaling–dependent manner (48). Thus, within the RVM^BDNF^ neurons, BDNF could determine the excitatory tone. While substantial insights on its role in strengthening synaptic transmission and plasticity associated with learning and memory have been obtained over decades of research since BDNF was first discovered (49, 50), the precise location, release, and functional importance of the various downstream signaling pathways elicited by BDNF in the pre- and postsynaptic membranes remain debated (20). Nonetheless, the numerous important functional roles of BDNF for synaptic function and behavior are undisputed (51).

Given the heterogeneity of underlying causes, clinical management of chronic pain remains a staggering challenge, and opioids remain essential analgesic options. However, the reliance on opioid analgesics has contributed to the opioid epidemic. Alternative therapeutic strategies that provide pain relief across different pain conditions are urgently needed (52). To make progress along this line of research, we have discovered the molecular identity of RVM neurons that regulate morphine-induced mechanical antinociception and identified BDNF/TrkB as essential components modulating neurotransmission.

## Materials and methods

### Animals

All procedures were performed with the approval of the northern Stockholm ethical committee (Stockholms norra djurförsöksetiska nämnd, Jordbruksverket). Arc-Cre^ERT2^ mice [B6.129(Cg)-*Arc*^*tm1.1(cre/ERT2)Luo*^/J, #021881, The Jackson Laboratory] were crossed to either the Cre-dependent tdTomato Ai9 mouse strain [B6.Cg-Gt(ROSA)26Sortm9(CAG-tdTomato)Hze/J, #007909, The Jackson Laboratory] or the Fos-TVA mouse strain (29). Cre^ERT2^ was induced in these mouse lines through a single intraperitoneal injection of 50 mg/kg 4-OH-tamoxifen approximately 60 minutes after stimulation. Vglut2-cre mice [Slc17a6tm2(cre)Lowl/J, #016963] and BDNF-2A-Cre mice [B6.FVB-Bdnfem1(cre)Zak/J, #020189] were purchased from the Jackson Laboratory. Both male and female mice were used in all experiments involving genetically modified mice and were randomly assigned to experimental groups. Behavioral tests were conducted by two independent experimenters blinded to each other. Experimenters were not blinded to experimental treatment. The Gal-Cre transgenic line was obtained from the Mutant Mouse Resource and Research Centers (MMRRC, Stock# 031060-UCD). Females were used for experiments involving only non-transgenic animals. Female WT mice (C57BL/6J, #000664) were purchased from the Jackson Laboratory and were used as a control. Mice were kept on a 12-hour day-night cycle and had access to food and water ad libitum.

### Chemicals

The following chemical compounds were used: 4′,6-diamidino-2-phenylindole (DAPI, D1306, Thermo-Fisher Scientific, Waltham MA, USA), clozapine (C6305, Sigma-Aldrich St. Louis MO, USA), salvinorin B (75250, Sigma-Aldrich), actinomycin D (A1410, Merck, Darmstadt, Germany), and 4-OH-tamoxifen (H6278, Sigma-Aldrich). Clozapine was resuspended in 100% ethanol to a stock concentration of 1 mg/ml and was further diluted in PBS. Clozapine (intraperitoneally, 0.3 mg/kg unless otherwise stated) was injected approximately 45 minutes before behavioral assessment. Salvinorin B was diluted in DMSO to a concentration of 10 mg/ml and approximately 20-30 µl (10mg/kg) was injected subcutaneously 15 minutes before behavioral assessment. Actinomycin D was resuspended in 100% ethanol at a stock concentration of 1.5 mM and further diluted in homogenization buffer before dissociation. 4-OH-tamoxifen was first solubilized in ethanol (10% final volume) and further diluted with corn oil. Morphine hydrochloride 20 mg/ml with 6.2 mg/ml NaCl in water, pH 4 to 6.5 (348375, APL, Huddinge, Sweden) was injected subcutaneously at a dose of 10mg/kg unless otherwise stated. Morphine was injected approximately 15 minutes before behavioral assessment.

### Viral injections (intracranial, intraspinal, intravenous)

Virus injections were made in 6- to12-week-old adult mice. Animals were given 5mg/kg carprofen subcutaneously 10 min before surgery. Animals were anesthetized with 2 to 5% isoflurane, and when immobilized, placed into a stereotaxic frame (David Kopf Instruments, Tujunga, CA, USA). Injections were performed with a pulled glass capillary (internal diameter approximately 30 to 40 µm) attached to a 10 µl Hamilton syringe.

### Intraspinal injections

The vertebral column was fixed using a pair of spinal adapters. After carefully removing the paraspinal muscles the T13 dorsal spinous process was removed to expose the dura mater and lumbar spinal cord at L3 and L4. Viral vectors were injected 300 μm to the right of the posterior median spinal vein at a depth of 250 μm. Pulled glass micropipettes were used to inject 250nl of vector solution at a speed of 50 nl/min. Injections (3 × 250 nl) were spaced approximately 1mm apart. The capillary was kept in the injection site for 5 min after the infusion was complete and then retracted slowly. Wounds were sutured and the animals were placed on a heating pad and monitored until fully recovered. On average spinal injections lead to viral transduction of neurons located in ipsilateral spinal laminae I to IV in most animals. Animals were given at least 10 days of recovery period. For Fos-TVA*Arc-Cre^ERT2^ experiments, mice were injected with either PBS or clozapine approximately 2 hours before virus injection to allow for sufficient expression of TVA in stimulated cells.

### Intracranial injections

The skulls of anesthetized mice were shaved, cleaned with chlorhexidine and fixed in place with ear bars. An incision of approximately 1cm was made in the skin covering the skull, and the skull position was adjusted until bregma and lambda were level. A small hole was drilled on the midline approximately 5mm caudal to bregma, and a pulled glass capillary filled with viral solution was slowly lowered approximately 4.8mm into the brain. Virus solution (300 nl) was injected at approximately 60 nl/min. The capillary was allowed to remain in place for 5 minutes after injection to ensure proper diffusion of the injected solution. The capillary was slowly retracted, and the skin was sutured. Animals were allowed to recover on a heating pad and were given at least 10 days to recover from surgery.

### Intravenous injections

Adult mice were placed in a restrainer (Agnthos, Lidingö, Sweden) and 5% xylocaine cream was applied to the tail approximately 15 minutes before injection. The tail was soaked in warm water to dilate the vein, and 100 µl AAV [1 x 10^11^ viral genomes (vg) per animal] was injected with a 27-gauge needle. The needle was allowed to remain in place for approximately 15 s to prevent leakage and the tail was briefly compressed to stanch bleeding.

### Virus

The following viruses were purchased from the Viral Vector Facility (VVF) at University of Zurich (UZH): AAVretro-CAG-EGFP-WPRE-SV40p(A) (v24, 5 x 10^12^ vg/ml), AAV5-hSyn1-dlox-hM3D(Gq)_mCherry(rev)-dlox (v89, 6 x10^12^ vg/ml), AAV9-hSyn1-dlox-HA_hKORD_IRES_mCitrine(rev)-dlox (v94, 6 x 10^12^ vg/ml), AAVretro-hSyn1-chl-dlox-EBFP2_2a_NLS_FLPo(rev)-dlox (v175, 5.1 x 10^12^ vg/ml), AAV5-hSyn1-dFRT-hM3D(Gq)_mCherry(rev)-dFRT (v189, 3.5 x 10^12^ vg/ml), AAV5-hSyn1-dFRT-HA_hKORD_IRES_mCitrine(rev)-dFRT (v191, 3.2 x 10^12^ vg/ml), AAV9-hEF1a-DIO-hM3D(Gq)-mCherry (v98, 5.2 x 10^12^ vg/ml), AAV9-hEF1a-DIO-hM4D(Gi)-mCherry (v104, 1 x 10^13^ vg/ml), AAV9- hSyn1-chl-dlox-oCHIEF_tdTomato(rev)-dlox-WPRE-SV40p(A) (v463, 8.2 x 10^12^ vg/ml), and AAVPHP.eB-hCMV-HBbl/E-dlox-TVA_mCherry_2A_oG(rev)-dlox-hGHp(A) (v320, 1.1 x 10^13^ vg/ml). AAV9-GFP-U6-m-BDNF-shRNA, AAV9-GFP-U6-scrambled-shRNA, AAV9-hSyn1-DIO-mCherry-mBDNF-shRNAmir, AAV9-hSyn1-DIO-mCherry-mNtrk2-shRNAmir, AAV9-hSYN-DIO-mCherry-scrmb-shRNAmir (all approximately 1 x 10^13^ vg/ml) were designed, validated, and manufactured by Vector Biolabs. AAV9-EF1a-DIO-BDNF-Flag-pHluorin-WPRE-bGHpA and AAV9-nEF1a-DIO-EGFP-WPRE-bGHpA (both approximately 2 x 10^12^vg/ml) were purchased from BrainVTA. AAV9-CAG-flex-WGA (2 x 10^13^ vg/ml) was purchased from Neurophotonics (Canadian Neurophotonics Platform Viral Vector Core Facility, RRID:SCR_016477) and EnvA-pseudotyped pLV-hSyn-KORD-GFP (2.5 x 10^9^ vg/ml) was custom designed and prepared by the Viral Vector Core at Duke University School of Medicine. EnvA-pseudotyped SAD deltaG eGFP (5 x 10^7^ focus-forming units/ml) was generously provided by K. K. Conzelmann (LMU Munich).

The sequences for the shRNAs used are as follows: shBDNF: GCT GAAGTGTACAAGTCCGCGTCCTGTTTTGG CCAC TGACTGACAGGACGCGCTTGTACACTT CAG, shTrkB: GCT GTCAAGGTGGCGGAAATGTCTCGTTTTGGCCACTGACTGACGAGACATTCGCCACCT TGA CAG

### Immunofluorescence/quantification

#### Tissue preparation and cryosectioning

Mice were anesthetized with isoflurane and transcardially perfused with 0.1M PBS. For immunohistochemical experiments, mice were then perfused with 4% paraformaldehyde (PFA) before removal of brains and/or spinal cords. Brains and spinal cords were post-fixed for 18 and 2 hours, respectively, before dehydration in 30% Sucrose-PBS. Following dehydration, tissue samples were frozen in optimal cutting temperature (OCT) compound and kept at -80°C until cryosectioning. For fluorescence *in situ* hybridization experiments, brains and/or spinal cords were removed immediately after perfusion with PBS, snap frozen in OCT compound and stored at -80°C. Tissue was sectioned at a thickness of 20µm and sections were stored at -80°C before staining.

#### Immunohistochemsitry

Cryosections were allowed to dry at 37°C for 30 minutes, followed by two 5-minute washes with 0.1M PBS. Sections were permeabilized with 0.1% Tween-20 diluted in PBS (PBS-T) for 15 minutes. Following permeabilization, sections were blocked for 1 hour with 5% BSA diluted in PBS. Following the blocking step, primary antibodies were diluted in 5% BSA, and applied to the slides overnight at 4°C. The next day, the primary antibody was removed, and sections were washed three times (5 min each) with PBS. The appropriate secondary antibody was diluted in PBS and applied to the sections for 1 hour at room temperature. Following removal of the secondary antibody, slides were washed three times with PBS, and DAPI was applied at a concentration of 0.01 mg/ml for 5 min. The DAPI solution was removed, and slides were mounted with fluorescence mounting medium (Agilent, Santa Clara, CA, USA). The following primary antibodies were used: rabbit anti-lectin (WGA, T4144, Sigma-Aldrich, 1:2000), biotinylated rabbit anti-RFP (600-406-379, Rockland, 1:500), mouse anti-NeuN (MAB377, Sigma-Aldrich, 1:1000), mouse anti-Arc (C-7, Santa Cruz Biotechnology, 1:2000), rabbit anti-galanin (a gift from Tomas Hökfelt, 1:400). The following secondary antibodies were used: Alexa-488/555 donkey anti-rabbit IgG A-21206/A-31572), Alexa488/647 donkey anti-mouse IgG (A-21202/A-31571), and streptavidin Alexa555 conjugate (S32355). All secondary antibodies were purchased from Thermo-Fisher Scientific and used at a dilution of 1:1000. All sections were counterstained with DAPI.

#### Fluorescence in situ hybridization

Fluorescence *in situ* hybridization was performed using the RNAscope Multiplex Fluorescent Detection Reagents v2 kit (Biotechne, Minneapolis MN, USA), according to the manufacturer’s instructions for fresh frozen tissue. The following probes were used: Slc32a1(#319191), Slc17a6 (#319171), Tph2 (#318691), Syp (#426521), Sst (#404631), Ecel1 (#475331), Pdyn (#318771), Nkx2-2 (#551981), Nptx2 (#316901), Myo3b (#871551), Gli3 (#445511), Tacr3 (#481671), Cdh23 (#567261), Hcrtr2 (#460881), Pax8 (#574431), Hmcn1 (#834921), Gad1 (#400951), Pde11a (#481841), Atp2b4 (#480861), Tcf7l2 (#466901), Syt17 (#495741), Phldb2 (#871471), Ebf2 (#550841), Gabra1 (#435351), Gria3 (#426251), Bdnf (#457761), Fos (#316921), Gal (#400961), EGFP (#400281), pFlpO (#520791), Oprm1 (#315841), mCherry (#431201), and vector-EF1a-mCitrine-T1 (#1040671). Opal fluorophores were purchased from Akoya Biosciences (Marlborough MA, USA) and were used at a dilution of 1:1500. All sections were counterstained with DAPI.

#### Quantification

Images of stained sections were captured using a Zeiss LSM700 microscope (Zeiss, Oberkochen, Germany) and analyzed with Zen 2010 software (Zeiss). For quantification of stained sections, at least two to three sections per animal were analyzed throughout the rostro-caudal extent of the RVM or lumbar spinal cord. At least three animals were used for each experimental condition. Arc staining required antigen-retrieval and could not be performed together with NeuN staining, hence for Arc;RFP and RFP;NeuN staining, quantification was conducted on adjacent sections. Blinding to experimental conditions was not performed.

### Behavior

#### Chemogenetics

For activation of hM3D(Gq)-expressing neurons, 0.3 mg/kg clozapine (Sigma-Aldrich) was injected intraperitoneally approximately 45 minutes before behavioral testing. For inhibition of hKORD-expressing neurons, 10 mg/kg salvinorin B (Sigma-Aldrich) was injected subcutaneously 15 minutes before behavioral testing.

#### Von Frey

Mice were placed in glass cups on an elevated wire grid and the plantar surface of the hind paw was stimulated with a set of calibrated von Frey filaments (0.008 to 2g). After a 30 min resting period on a mesh floor, the plantar surface of the hind paw was stimulated with a series of von Frey monofilaments (Stoelting, IL, USA) with increasing force until the responses were elicited. The 50% paw withdrawal threshold was determined using Dixon’s up-down method. For animals with response thresholds greater or less than detectable limits (>2g or <0.03g), the maximum or minimum value (2 or 0.03g, respectively) was recorded. For noxious mechanical pricking stimulation, a 1 g von Frey filament was applied on each paw two times with a gap of 1 to 2 minutes. Paw licking and flinching behaviors were noted as response and reported as nocifensive behavior. Nocifensive response time was measured manually using a stopwatch for 1 minute after stimulation.

#### Hargreaves

Thermal withdrawal latency was established using Hargreaves thermal test. Mice were placed in a clear plastic chamber above a glass floor. A radiant heat source (IITC, Woodland Hills, CA) was placed below the floor and used to illuminate the hind paw of the test animal. The time to withdrawal of the affected paw was recorded, and each paw was stimulated three times with a 2-minute interval between stimulations.

#### TRAPing of morphine- and heat-responsive neurons

For capturing morphine activated neurons, Arc-Cre^ERT2^ mice were injected subcutaneously with 10 mg/kg morphine. After 75 minutes, mice were injected intraperitoneally with 50 mg/kg 4-OH-tamoxifen. To capture neurons activated by noxious heat, one hind paw was stimulated with the Hargreaves device five times until withdrawal was observed. The mice were allowed to rest for 10 minutes before receiving the stimulus again on the same paw. This procedure was repeated three more times, and after an additional 20 minutes mice were injected intraperitoneally with 50 mg/kg 4-OH-tamoxifen.

#### Hot plate

Nocifensive behaviors in response to noxious thermal stimulation were conducted using the hot plate test. Mice were placed in a square clear-plastic chamber (16 cm by 16 cm) directly on top of a hot plate (Bioseb, France) set to 48°C. Mice were recorded for 2 minutes, and numbers of nocifensive responses (licking, biting, shaking) were quantified. Following the recording period, mice were quickly removed from the hot plate and allowed to recover for at least 24 hours.

#### Carrageenan

λ-Carrageenan (HY-N9470, MedChemExpress, NJ, USA) was dissolved in PBS to obtain a 1% solution. Approximately 15 µl was injected into one hind paw using a 50 µl Hamilton syringe by intraplantar injection, and the mice were allowed to recover for 48 hours. Mechanical thresholds were then determined using von Frey filaments and the up-down method as described above.

### Single-cell and single-nucleus RNA-sequencing

#### Preparation of nuclei suspensions

Single nucleus suspensions were prepared from isolated RVM and spinal cord samples by means of dounce homogenization in Tris buffer containing 0.1% Triton X-100, as well as protease and RNAse inhibitors, and where appropriate, actinomycin D at a concentration of 15 µM. Debris was removed by gradient centrifugation. To obtain enriched neuronal samples, nuclei suspensions were subjected to fluorescence-activated cell sorting (FACS) for either NeuN (Figs. 2A and 5A), or tdTomato (Fig. 2F). FACS was performed using a BD FACSAria III Sorter (BD Biosciences, Franklin Lakes, NJ USA). Nuclei were captured, and libraries were prepared using 10x Genomics Chromium Single Cell kit v3.0 according to the manufacturer’s instructions. Sequencing was performed at the National Genomics Infrastructure (NGI) Sweden with the Illumina NovaSeq 6000 system.

#### Preparation of cell suspensions

Single-cell suspensions were prepared from WT mice previously injected intraspinally with AAVretro-EGFP to label descending projections from the RVM. The RVM was then sectioned by Vibratome and gently dissociated with papain and deoxyribonuclease. Neurons were enriched and debris was removed by gradient centrifugation. Single EGFP-positive cells were selected through FACS. Single cells were captured, and libraries were prepared using 10x Genomics Chromium Single Cell kit v2.0 according to the manufacturer’s instructions. Sequencing was performed using the Illumina HiSeq 2500 platform. FACS was performed as described above.

#### Data Analysis

The CellRanger (version 7.07.1, https://www.10xgenomics.com/support/software/cell-ranger/latest) pipeline and Mouse transcriptome version mm10 were used to align reads and generate gene expression data. R (version 4.1.0, https://www.r-project.org/) and Seurat (version 4.3.0) were used to analyze single-cell and single-nucleus sequencing data. For the reference dataset, a total of 13,305 nuclei were obtained from three whole RVM samples originating from 21 mice. Initial quality control and preliminary clustering was performed as per the guided clustering tutorial available on the Satija lab’s website (https://satijalab.org/seurat). Data were filtered by gene expression for neuronal markers (Meg3, Rbfox3, Snap25) to remove non-neuronal nuclei. To remove neuronal nuclei from regions outside the RVM, gene expression was compared with *in situ* data from the Allen Mouse Brain Atlas; clusters enriched for genes located outside the RVM were removed from the analysis. After filtering, 6631 nuclei remained, with an average of 4030 genes detected per nucleus. For sequencing of nuclei from Arc-Cre^ERT2^;R26-Tomato mice, nuclei were clustered as above, and 1376 “No Stimulus” and 504 “Morphine” nuclei remained with an average gene expression of approximately 5850 and 6300 genes per nucleus, respectively. Cluster identities for these nuclei were determined with a classifier trained on the reference dataset. Briefly, the classifier was first trained with scPred (53) using a mixture discriminant analysis (MDA) model. After label predictions, clusters that were not found to be present in all three samples were removed from the final analysis. For sequencing of descending RVM cells, cells were clustered as described above, and 68 cells were used with an average gene expression of approximately 4800 genes per cell. Cluster identity was predicted as described above. For spinal cord samples, nuclei were clustered and cell types identified using the same approach as before, but this time using the spinal cord dorsal horn atlas described in Zhang et al (24) as reference data. In total, 12903 “WT” nuclei, 6843 “Gq + shScr” nuclei, and 8859 “Gq + shBDNF” nuclei remained with an average of 2635, 1572, and 1523 genes expressed per nucleus, respectively. Neuronal activity was determined by examining the expression 103 IEGs as previously reported (23). The Seurat function AddModuleScore was used to give an assessment of the average IEG expression as compared with background gene expression. “IEG positivity” was determined by counting the number of nuclei with an enrichment in IEG expression (IEG score of > 0) and expressing this as a percentage of total nuclei per cluster. Changes in this percentage as compared with WT were interpreted as an increase or decrease in neuronal activity. In Fig. 5B, “IEG score” is presented as the IEG Module Score + 0.05 for all samples. Blinding to experimental conditions was not performed.

#### Enrichment analysis

A significant feature (neuronal activity) was detected by comparing the expression of feature-related genes as described above, 103 IEGs (23) with the expression of randomly selected background genes. Briefly, we first identified the most variable genes, then selected top marker genes for each cell type and merged them with the most variable genes to build a candidate gene pool. Next, feature-related genes were selected from the candidate gene pool for feature scoring, and the remaining genes were used as the background gene pool. The background genes were ranked by their expression and divided into 50 intervals. Fifty genes from each interval were randomly selected and merged to form a random background gene matrix. The feature scores were calculated by comparing the differential average expression between the feature (neuronal activity) genes and the randomized background genes. To evaluate the false discovery rate (FDR), we repeated the above process 100 times. The cell was considered neuronally active if the differential average expression was >0 and the FDR *P* value was <0.01. To reduce the bias from the number of top marker genes, we evaluated using three thresholds: top 250, top 500, and top 1000 marker genes. For the top 250 marker genes, we had 2629 genes in the gene pool, 20 of which were neuronal activity genes, defining 8361 active neurons in total. For the top 500 marker genes, there were 4366 genes in the gene pool, 34 of which were neuronal activity genes, defining 7677 active neurons in total. And for the top 1000 marker genes, there were 11,439 genes in the gene pool, 76 of which were neuronal activity genes, defining 6274 active neurons in total. Lastly, the neuronal activity of a cell type was considered robustly perturbed between control and treated groups only if the cell type's neuronal activity consistently differed in all three evaluations.

#### FAST (LED exposure + FAST recording)

Mice previously injected with AAV-dlox-oChIEF-dlox were anesthetized with isoflurane and placed in a stereotactic frame. The spinal column surrounding the T13 region was exposed and stabilized with spinal adapters. The T13 vertebra was removed to expose the spinal cord and a microelectrode array (MEA) was inserted into the L4-L5 region, approximately 350µm deep. As previously described (19), glutamate dynamics were assessed by MEA recordings with two electrode sites treated with L-glutamate oxidase, which breaks down L-glutamate into α-ketoglutarate and hydrogen peroxide (H2O2). By applying fixed potential, the H_2_O_2_ was oxidized, and the resulting electron loss and current was recorded using a Fast Analytical Sensing Technology-16 (FAST-16 MKII) electrochemistry instrument (Quanteon, Nicholasville, KY USA). oChIEF-expressing axon terminals were stimulated by applying a 2 second pulse of blue light (470nm) directly to the exposed spinal cord at a distance of approximately 5mm. Modulating the LED strength for fig. S7 was accomplished by increasing the distance from the tissue to approximately 25 mm. A MATLAB graphic interface was then used to calculate glutamate concentrations from an average of four to six amplitudes per mouse. Maximum amplitude of evoked glutamate (Glu Amplitude) and the time for 80% decay from maximum amplitude (T80) were measured.

### Anterograde and retrograde tracing

#### Anterograde WGA tracing

Mice were injected intracranially in the RVM with a virus encoding Cre-dependent anterograde tracer WGA. After 7 days, the mice were transcardially perfused with PFA and the lumbar spinal cord was removed and sectioned. Cryosections were stained using antibodies against Lectin/WGA (1:1000) and galanin (1:400) as described above.

#### Monosynaptic rabies tracing

Mice were treated by intravenous injection with an AAV encoding Cre-dependent TVA and oG protein, and 3 weeks were allowed for sufficient expression. EnvA-pseudotyped rabies virus was then injected into the lumbar spinal cord, and an additional 10 days were allowed to ensure sufficient retrograde transport and expression of viral proteins. Mice were transcardially perfused with PFA, and the brain and lumbar spinal cord were collected and cryosectioned as described above.

#### Statistics

Data were analyzed using GraphPad Prism 9 (GraphPad Software, Boston, MA, USA) and expressed as mean ± standard error of the mean (SEM). Statistical significance was calculated with one- or two-way analysis of variance using the Holm-Šídák multiple comparison test or using the unpaired or paired Student’s t-test where appropriate. Degree of significance was represented as follows: *P ≤ 0.05, **P ≤ 0.01, and ***P ≤ 0.001. Further details on statistics are available in the supplementary statistics file.

## Supplementary Material

Fig. S1-8

Supplementary Statistics File

## Data Availability

Raw and processed data are available in the NCBI Gene Expression Omnibus, under accession number GSE253816. All remaining data are available in the manuscript or the supplementary materials. An interactive web resource for browsing the RVM dataset is available at https://ernforslab.shinyapps.io/MouseRVM/

## References

[1] Corder G, Castro DC, Bruchas MR, Scherrer G (2018). Endogenous and Exogenous Opioids in Pain. Annu Rev Neurosci.

[2] Ossipov MH, Dussor GO, Porreca F (2010). Central modulation of pain. J Clin Invest.

[3] Oliveras JL, Redjemi F, Guilbaud G, Besson JM (1975). Analgesia induced by electrical stimulation of the inferior centralis nucleus of the raphe in the cat. Pain.

[4] Proudfit HK, Anderson EG (1975). Morphine analgesia: blockade by raphe magnus lesions. Brain Res.

[5] Basbaum AI, Clanton CH, Fields HL (1976). Opiate and stimulus-produced analgesia: functional anatomy of a medullospinal pathway. Proc Natl Acad Sci U S A.

[6] Gomtsian L (2018). Morphine effects within the rodent anterior cingulate cortex and rostral ventromedial medulla reveal separable modulation of affective and sensory qualities of acute or chronic pain. Pain.

[7] Zhuo M, Gebhart GF (1997). Biphasic modulation of spinal nociceptive transmission from the medullary raphe nuclei in the rat. J Neurophysiol.

[8] Fields HL, Basbaum AI, Heinricher MM, McMahon SB, Koltzenburg M (2006). Wall and Melzack’s Textbook of Pain.

[9] Cai Y-Q, Wang W, Hou Y-Y, Pan ZZ (2014). Optogenetic activation of brainstem serotonergic neurons induces persistent pain sensitization. Mol Pain.

[10] François A (2017). A Brainstem-Spinal Cord Inhibitory Circuit for Mechanical Pain Modulation by GABA and Enkephalins. Neuron.

[11] Jiao Y (2023). Molecular identification of bulbospinal ON neurons by GPER, which drives pain and morphine tolerance. J Clin Invest.

[12] Zhang Y (2015). Identifying local and descending inputs for primary sensory neurons. J Clin Invest.

[13] Xue Y (2024). Dissecting neural circuits from rostral ventromedial medulla to spinal trigeminal nucleus bidirectionally modulating craniofacial mechanical sensitivity. Prog Neurobiol.

[14] Fields HL, Heinricher MM (1989). Brainstem modulation of nociceptor-driven withdrawal reflexes. Ann N Y Acad Sci.

[15] Lau BK, Vaughan CW (2014). Descending modulation of pain: the GABA disinhibition hypothesis of analgesia. Curr Opin Neurobiol.

[16] Goodchild CS, Nadeson R, Cohen E (2004). Supraspinal and spinal cord opioid receptors are responsible for antinociception following intrathecal morphine injections. Eur J Anaesthesiol.

[17] Sakurai K (2016). Capturing and Manipulating Activated Neuronal Ensembles with CANE Delineates a Hypothalamic Social-Fear Circuit. Neuron.

[18] Tan CL (2016). Warm-Sensitive Neurons that Control Body Temperature. Cell.

[19] Hascup KN, Hascup ER, Pomerleau F, Huettl P, Gerhardt GA (2008). Second-by-second measures of L-glutamate in the prefrontal cortex and striatum of freely moving mice. J Pharmacol Exp Ther.

[20] Song M, Martinowich K, Lee FS (2017). BDNF at the Synapse: Why Location Matters. Mol Psychiatry.

[21] Lessmann V, Gottmann K, Heumann R (1994). BDNF and NT-4/5 enhance glutamatergic synaptic transmission in cultured hippocampal neurones. Neuroreport.

[22] Takei N (1998). Brain-derived neurotrophic factor induces rapid and transient release of glutamate through the non-exocytotic pathway from cortical neurons. J Biol Chem.

[23] Hrvatin S (2018). Single-cell analysis of experience-dependent transcriptomic states in the mouse visual cortex. Nat Neurosci.

[24] Zhang M-D, Kupari J, Su J, Hu Y, Magnusson KA, Calvo-Enrique L, Usoskin D, Albisetti GW, Leavitt AD, Zeilhofer HU, Hökfelt T (2024). Neural ensembles that encode affective mechanical and heat pain in mouse spinal cord. bioRxiv.

[25] Chan KY (2017). Engineered AAVs for efficient noninvasive gene delivery to the central and peripheral nervous systems. Nat Neurosci.

[26] Barbaro NM, Heinricher MM, Fields HL (1986). Putative pain modulating neurons in the rostral ventral medulla: reflex-related activity predicts effects of morphine. Brain Res.

[27] Barbaro NM, Heinricher MM, Fields HL (1989). Putative nociceptive modulatory neurons in the rostral ventromedial medulla of the rat display highly correlated firing patterns. Somatosens Mot Res.

[28] Fields HL, Bry J, Hentall I, Zorman G (1983). The activity of neurons in the rostral medulla of the rat during withdrawal from noxious heat. J Neurosci.

[29] Heinricher MM, Kaplan HJ (1991). GABA-mediated inhibition in rostral ventromedial medulla: role in nociceptive modulation in the lightly anesthetized rat. Pain.

[30] Heinricher MM, Morgan MM, Tortorici V, Fields HL (1994). Disinhibition of off-cells and antinociception produced by an opioid action within the rostral ventromedial medulla. Neuroscience.

[31] Maloney KJ, Mainville L, Jones BE (1999). Differential c-Fos expression in cholinergic, monoaminergic, and GABAergic cell groups of the pontomesencephalic tegmentum after paradoxical sleep deprivation and recovery. J Neurosci.

[32] Winkler CW (2006). Kappa Opioid Receptor (KOR) and GAD67 Immunoreactivity Are Found in off and neutral Cells in the Rostral Ventromedial Medulla. Journal of Neurophysiology.

[33] Otsu Y, Aubrey KR (2022). Kappa opioids inhibit the GABA/glycine terminals of rostral ventromedial medulla projections in the superficial dorsal horn of the spinal cord. J Physiol.

[34] Nguyen E (2022). Medullary kappa-opioid receptor neurons inhibit pain and itch through a descending circuit. Brain.

[35] Chen Q, Heinricher MM (2022). Shifting the Balance: How Top-Down and Bottom-Up Input Modulate Pain via the Rostral Ventromedial Medulla. Front Pain Res (Lausanne).

[36] Heinricher MM, McGaraughty S (1998). Analysis of excitatory amino acid transmission within the rostral ventromedial medulla: implications for circuitry. Pain.

[37] Huisman AM, Kuypers HG, Verburgh CA (1981). Quantitative differences in collateralization of the descending spinal pathways from red nucleus and other brain stem cell groups in rat as demonstrated with the multiple fluorescent retrograde tracer technique. Brain Res.

[38] Skagerberg G, Björklund A (1985). Topographic principles in the spinal projections of serotonergic and non-serotonergic brainstem neurons in the rat. Neuroscience.

[39] Ganley RP, Magalhaes de Sousa M, Ranucci M, Werder K, Öztürk T, Wildner H, Zeilhofer HU (2023). Descending GABAergic Neurons of the RVM That Mediate Widespread Bilateral Antinociception. bioRxiv.

[40] Ganley RP (2023). Targeted anatomical and functional identification of antinociceptive and pronociceptive serotonergic neurons that project to the spinal dorsal horn. eLife.

[41] Zeilhofer HU, Wildner H, Yevenes GE (2012). Fast Synaptic Inhibition in Spinal Sensory Processing and Pain Control. Physiol Rev.

[42] Häring M (2018). Neuronal atlas of the dorsal horn defines its architecture and links sensory input to transcriptional cell types. Nat Neurosci.

[43] Russ DE (2021). A harmonized atlas of mouse spinal cord cell types and their spatial organization. Nat Commun.

[44] Polgár E (2013). Functional differences between neurochemically defined populations of inhibitory interneurons in the rat spinal dorsal horn. Pain.

[45] Guo W (2006). Supraspinal Brain-Derived Neurotrophic Factor Signaling: A Novel Mechanism for Descending Pain Facilitation. J Neurosci.

[46] Lessmann V, Heumann R (1998). Modulation of unitary glutamatergic synapses by neurotrophin-4/5 or brain-derived neurotrophic factor in hippocampal microcultures: presynaptic enhancement depends on pre-established paired-pulse facilitation. Neuroscience.

[47] Tyler WJ (2006). BDNF increases release probability and the size of a rapidly recycling vesicle pool within rat hippocampal excitatory synapses. J Physiol.

[48] Li YX (1998). Expression of a dominant negative TrkB receptor, T1, reveals a requirement for presynaptic signaling in BDNF-induced synaptic potentiation in cultured hippocampal neurons. Proc Natl Acad Sci U S A.

[49] Linnarsson S, Björklund P, Ernfors P (1997). Learning deficit in BDNF mutant mice. Eur J Neurosci.

[50] Kang H, Schuman EM (1995). Long-lasting neurotrophin-induced enhancement of synaptic transmission in the adult hippocampus. Science.

[51] Wang CS, Kavalali ET, Monteggia LM (2022). BDNF signaling in context: From synaptic regulation to psychiatric disorders. Cell.

[52] Skolnick P, Volkow ND (2016). Re-energizing the Development of Pain Therapeutics in Light of the Opioid Epidemic. Neuron.

[53] Alquicira Hernandez J (2021). powellgenomicslab/scPred: Version 1.9.2.

