# Supplementary material for: Morphine-responsive neurons that regulate mechanical antinociception
Source: Science. Author manuscript; Available in PMC 2024 Sep 16. (PMC7616448; doi:10.1126/science.ado6593)
Supplement: Fig. S1-8 [file EMS198530-supplement-Fig__S1_8.pdf]

Science
AAAS

Supplementary Materials for

Morphine-responsive neurons that regulate mechanical antinociception

Michael Fatt, Ming-Dong Zhang†, Jussi Kupari†, Müge Altınkök, Yunting Yang, Yizhou Hu, Per Svenningsson, Patrik Ernfors*
*Corresponding author: patrik.ernfors@ki.se

The PDF file includes:

Figs. S1 to S8

Other Supplemental Materials for this manuscript include the following:

Supplementary Statistics File (separate file)

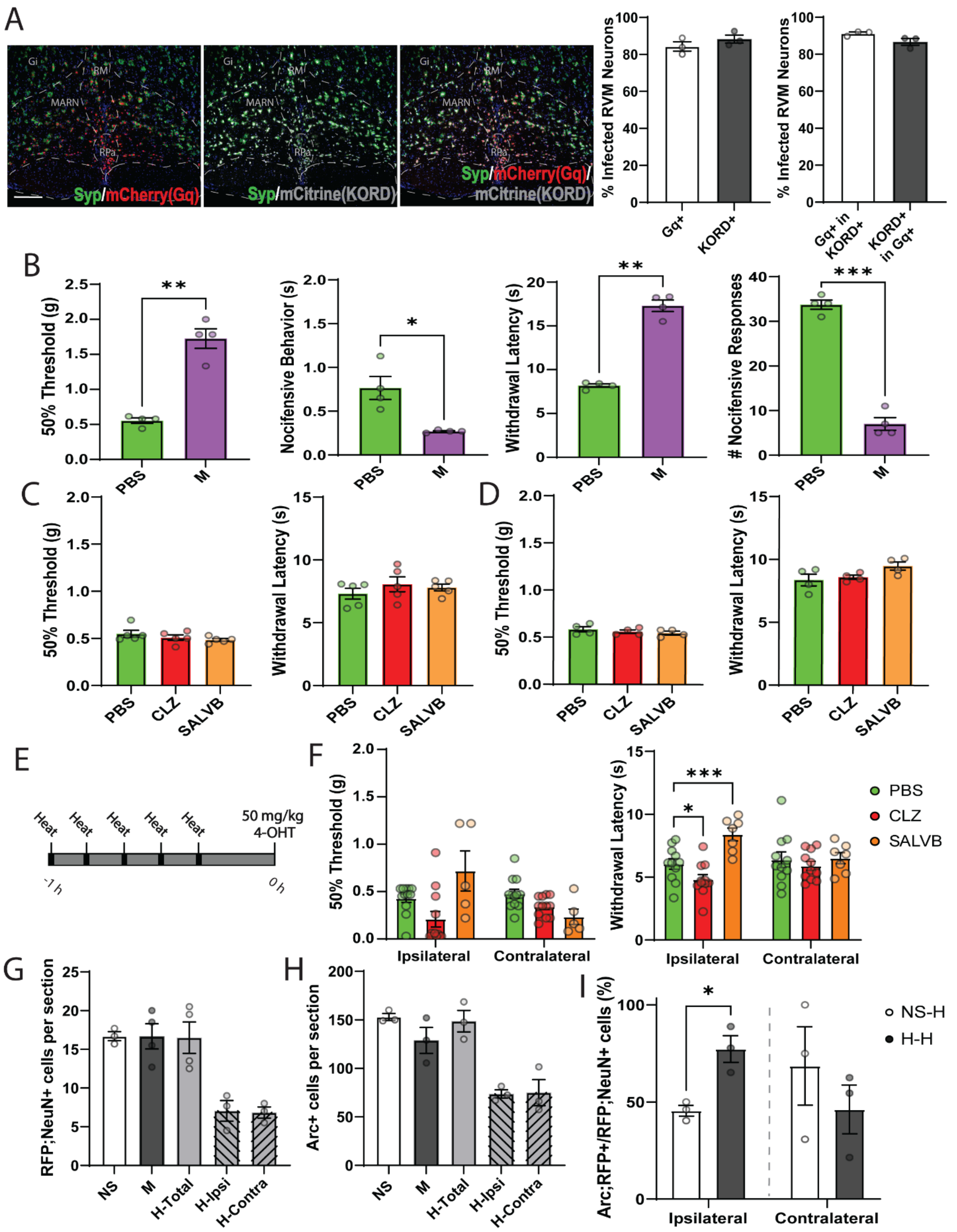
A
Gi
RM
MARN
RPa
Syp/mCherry(Gq)
Syp/mCitrine(KORD)
Syp/mCherry(Gq)/mCitrine(KORD)
% Infected RVM Neurons
Gq+
KORD+
Gq+ in KORD+
KORD+ in Gq+
B
50% Threshold (g)
Nocifensive Behavior (s)
Withdrawal Latency (s)
Nocifensive Responses
PBS
M
C
D
CLZ
SALVB
E
Heat
50 mg/kg 4-OHT
-1 h
0 h
F
Ipsilateral
Contralateral
PBS
CLZ
SALVB
G
RFP;NeuN+ cells per section
NS
M
H-Total
H-Ipsi
H-Contra
H
Arc+ cells per section
I
Arc;RFP+/RFP;NeuN+ cells (%)
NS-H
H-H

Figure S1: Capture and manipulation of heat responsive RVM neurons promotes hyperalgesia (related to Fig. 1).

A) *Left*, images of the RVM of a mouse co-injected with AAV-hM3D(Gq)-mCherry and AAV-hKORD-mCitrine showing Fluorescence *in situ* hybridization for pan-neuronal marker Synaptophysin (*Syp*, green), mCherry (red), and KORD (grey). White hatched lines indicate the different nuclei within/adjacent to the RVM. *MARN*: Magnocellular Reticular Nucleus, *RM*: Nucleus Raphe Magnus, *RPa*: Nucleus Raphe Pallidus, *Gi*: Gigantocellular Reticular Nucleus. Scale bar = 200µm. *Right*, Quantification of the proportion of neurons expressing either mCherry (Gq+) or mCitrine (KORD+), and the proportion of neurons with expression both markers. **B)** As seen in Fig. 1B, wild-type mice were injected with either PBS or Morphine, and pain behavior was assessed in response to non-noxious (*far left*) or noxious (*center left*) mechanical stimulation, or thermal stimulation via Hargreaves (*center right*, 35% IR intensity) or Hotplate (*far right*). **C)** Arc-Cre^{ERT2};R26-Tomato mice were injected in the RVM with AAVs expressing Cre-dependent hM3d(Gq) and hKORD, and neurons were captured in response to no stimulation. Mechanical (*left*) and thermal (*right*, Hargreaves, 35% IR intensity) withdrawal thresholds were then measured following either PBS, or activation (CLZ) or inhibition (SALVB) of the captured neurons. **D)** Wild-type mice were treated with either PBS, CLZ, or SALVB and mechanical (*left*) and thermal (*right*, Hargreaves, 35% IR intensity) withdrawal thresholds were measured. **E)** Schematic outline for the capture of RVM neurons activated in response to heat pain. 'Heat' was applied to one hind paw using the Hargreaves device until withdrawal was observed. **F)** Assessment of mechanical (*left*) and thermal (*right*) withdrawal thresholds in response to vehicle (PBS), or chemogenetic activation (CLZ) or inhibition (SALVB) of the RVM heat pain ensemble. *Left*, 50% withdrawal threshold in response to mechanical stimulation with von Frey filaments. *Right*, withdrawal latency in response to thermal stimulation as measured by the Hargreaves test (35% IR intensity). n ≥ 5 mice per condition, *** $p < 0.001$. **G-I)** Arc-Cre^{ERT2};R26-Tomato mice were subject to either no stimulus (NS), morphine (M), or heat pain (H) and coronal sections through the RVM were immunostained for endogenous Arc protein and Tomato (anti-RFP antibody). Quantification of the number of cells per section positive for Tomato (**G**), endogenous Arc protein (**H**), or double-positive (**I**, represented as proportion of total Tomato-positive RVM neurons). Arc;RFP and RFP;NeuN were quantified on adjacent sections. n ≥ 3 mice per condition, * $p < 0.05$. In the above figure, the data for **H** has been split into 'ipsilateral' and 'contralateral' to highlight the unilateral nature of the stimulus.

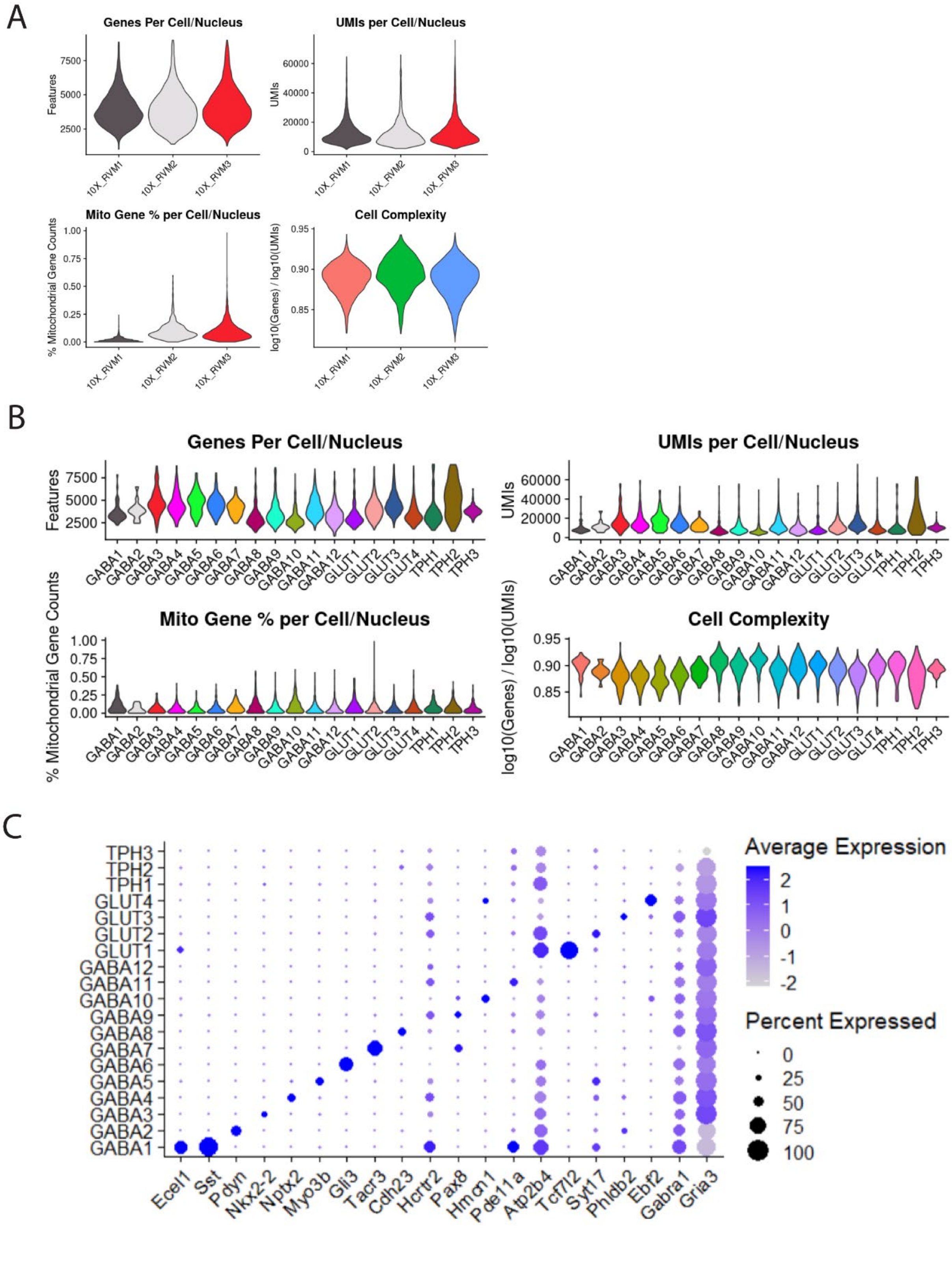
A
Genes Per Cell/Nucleus
Features
7500
5000
2500
10X_RVM1
10X_RVM2
10X_RVM3
UMIs per Cell/Nucleus
UMIs
60000
40000
20000
0
Mito Gene % per Cell/Nucleus
% Mitochondrial Gene Counts
1.00
0.75
0.50
0.25
0.00
Cell Complexity
log10(Genes) / log10(UMIs)
0.95
0.90
0.85
B
Genes Per Cell/Nucleus
UMIs per Cell/Nucleus
Mito Gene % per Cell/Nucleus
Cell Complexity
GABA1 GABA2 GABA3 GABA4 GABA5 GABA6 GABA7 GABA8 GABA9 GABA10 GABA11 GABA12 GLUT1 GLUT2 GLUT3 GLUT4 TPH1 TPH2 TPH3
C
TPH3 TPH2 TPH1 GLUT4 GLUT3 GLUT2 GLUT1 GABA12 GABA11 GABA10 GABA9 GABA8 GABA7 GABA6 GABA5 GABA4 GABA3 GABA2 GABA1
Ecel1 Sst Pdyn Nkx2-2 Nptx2 Myo3b Gli3 Tacr3 Cdh23 Hcrtr2 Pax8 Hmcn1 Pde11a Atp2b4 Tcf7l2 Syt17 Phldb2 Ebf2 Gabra1 Gria3
Average Expression
2
1
0
-1
-2
Percent Expressed
0
25
50
75
100

Figure S2: Quality control data for snRNA-seq and cluster-specific markers used for Fluorescence *in situ* hybridization (related to Fig. 2).

A, B) Quality control data for sequenced nuclei obtained from the mouse RVM. The graphs show average genes detected per nucleus (reported as features, *top left*), average Unique Molecule Identifiers (UMIs) per nucleus (*top right*), average mitochondrial genes detected per nucleus (*bottom left*), and average complexity (*bottom right*). Data is shown separated by sample (**A**) and by cell type (**B**). **C)** Dot plot showing the specificity of the markers/marker combinations used for detection and validation of unique clusters via fluorescence *in situ* hybridization.

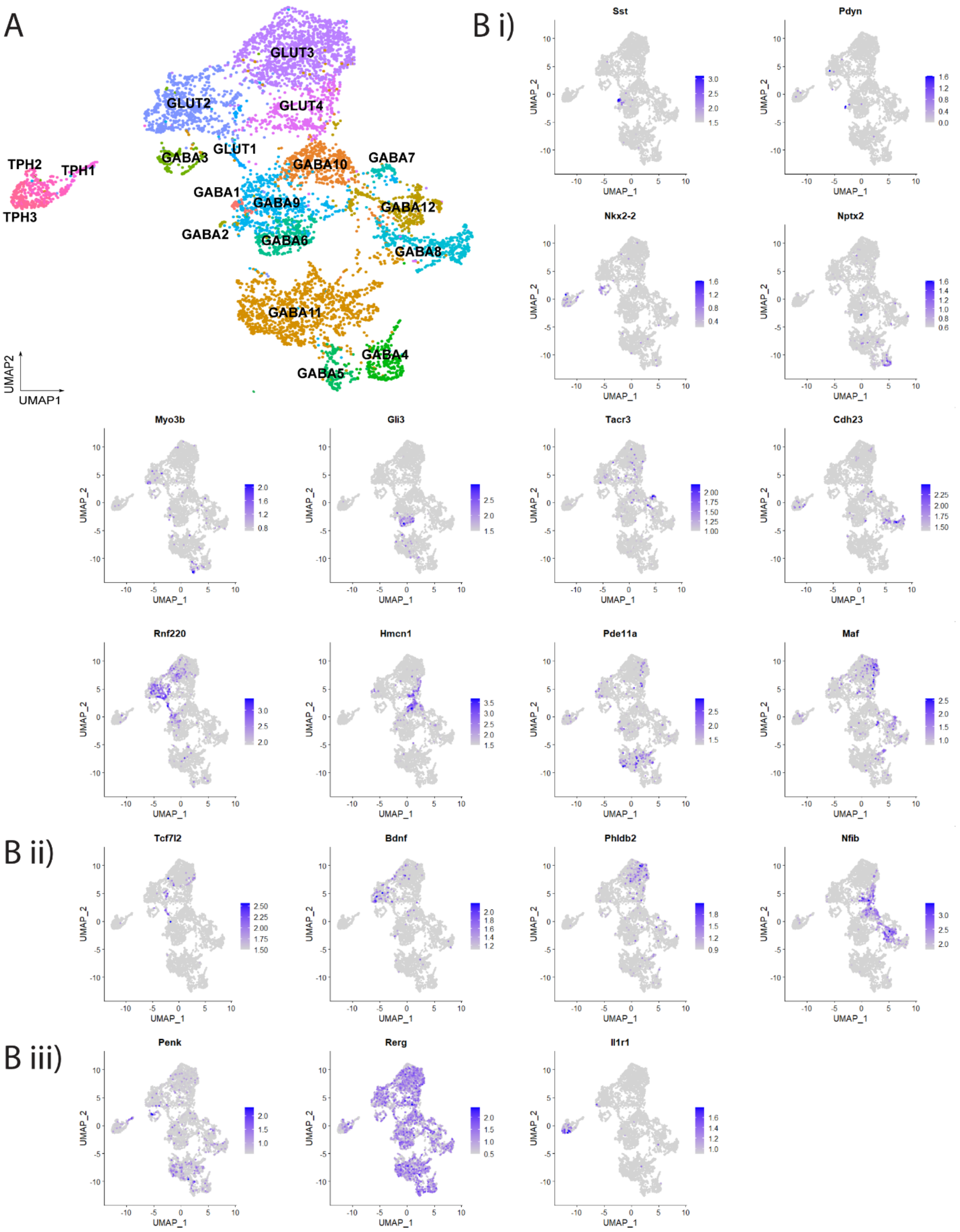
A
GLUT3
GLUT2
GLUT4
GLUT1
GABA3
GABA10
GABA7
TPH2
TPH1
TPH3
GABA1
GABA9
GABA12
GABA2
GABA6
GABA8
GABA11
GABA4
GABA5
UMAP2
UMAP1
B i)
Sst
Pdyn
Nkx2-2
Nptx2
Myo3b
Gli3
Tacr3
Cdh23
Rnf220
Hmcn1
Pde11a
Maf
B ii)
Tcf7l2
Bdnf
Phldb2
Nfib
B iii)
Penk
Rerg
Il1r1
UMAP_1
UMAP_2

Figure S3: Expression of markers used for identification of unique clusters (related to Fig. 2).

A) UMAP from Fig. 2A showing the clustering of RVM neurons. **B)** Feature plots showing the expression of individual markers used for *in situ* hybridization validation of unique GABAergic (*i*), Glutamatergic (*ii*), and Serotonergic (*iii*) clusters.

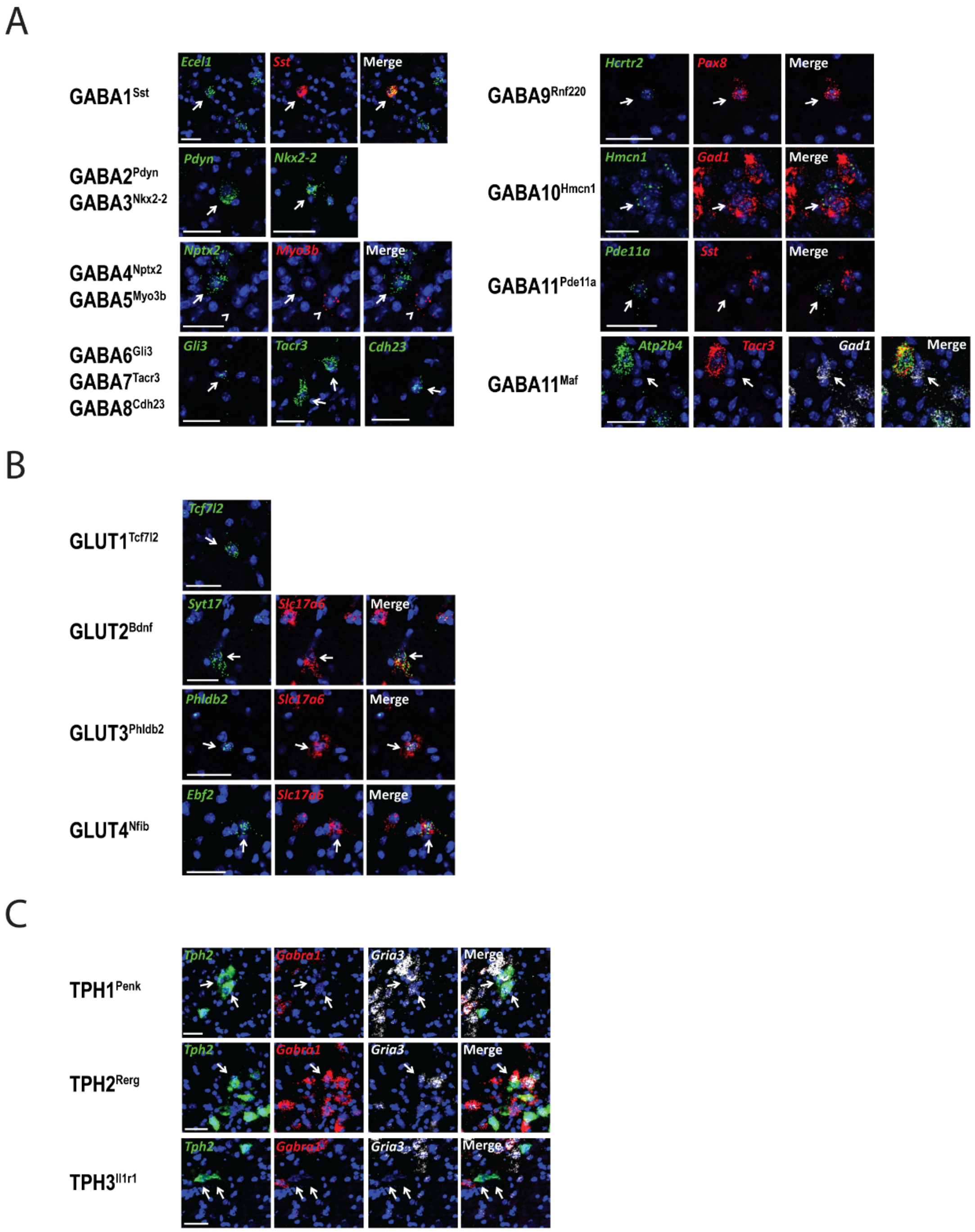

Figure S4: Fluorescence *in situ* validation of 19 unique neuronal clusters in the RVM (related to Fig. 2).

A-C) Fluorescence *in situ* hybridization validation of cell types using specific marker gene combinations confirmed the 19 unique clusters identified by snRNA-seq. Uncropped images from Fig. 2C are shown and separated into GABAergic (**A**), Glutamatergic (**B**), and Serotonergic (**C**) subtypes. Arrows denote marker-positive cells, and the arrowhead shows a *Myo3b*-positive cell. Scale bar = 40µm.

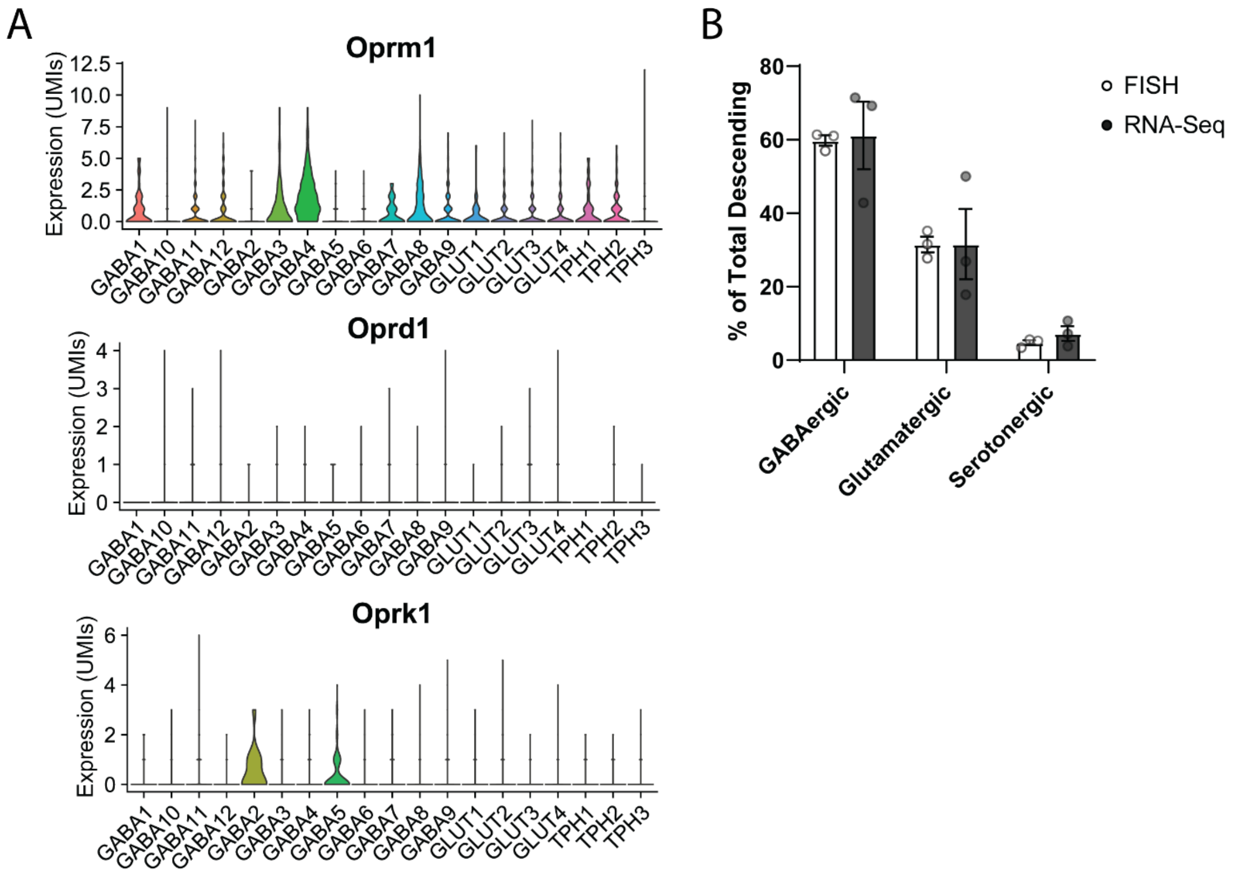

Figure S5: Opioid receptor expression and identification of descending RVM neurons by fluorescence *in situ* hybridization (FISH) and scRNA-Seq (related to Fig. 2-3).

A) Violin plots showing the expression of opioid receptor genes Oprm1 (*top*), Oprd1 (*middle*), and Oprk1 (*bottom*) in the 19 identified RVM neuronal populations.

B) Assessment of the proportion of descending RVM neurons that are GABAergic, Glutamatergic, or Serotonergic as identified *in vivo* (by FISH) or *in silico* (using the RNA-Seq data). $n \geq 3$ mice per condition.

A

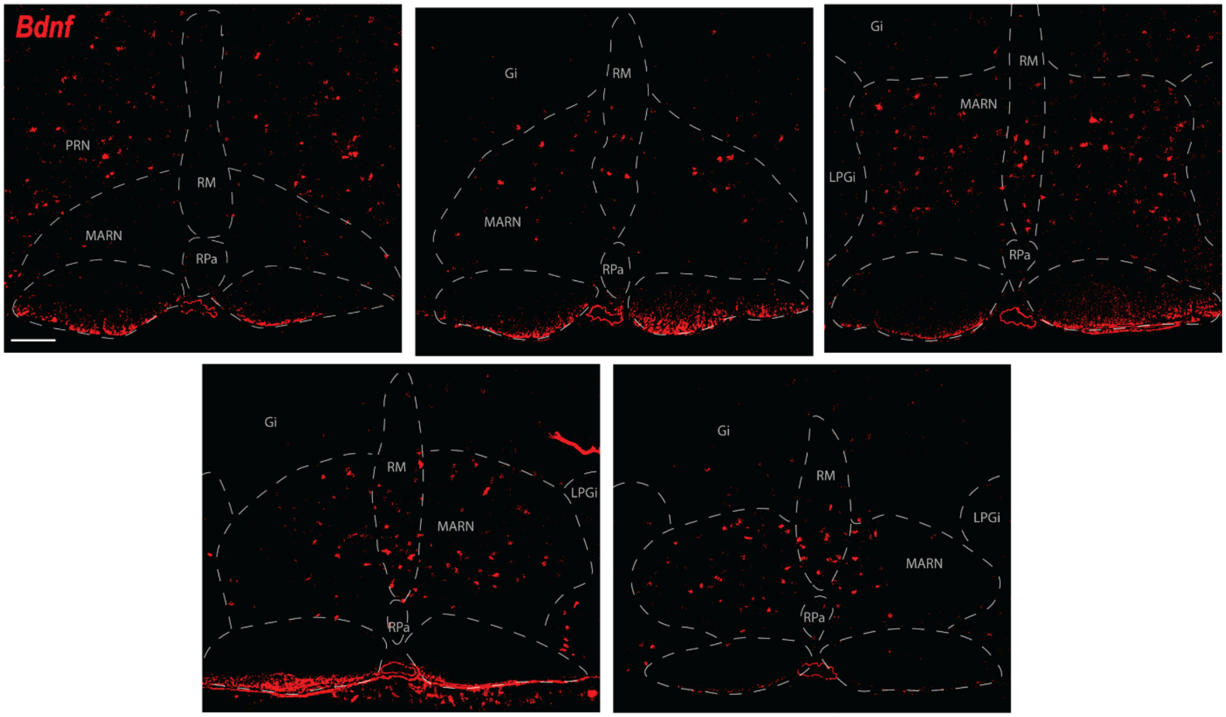

Figure S6: RVM^{BDNF} neurons are located throughout the rostro-caudal extent of the RVM (related to Fig. 4).

A) Fluorescence *in situ* hybridization for *Bdnf*. Images were taken throughout extent of the RVM, from rostral (*top left*) to caudal (*bottom right*). Images were spaced approximately 200µm apart. White hatched lines indicate the different nuclei within/adjacent to the RVM. Scale bar = 200µm. *PRN*: Pontine Reticular Nucleus, *MARN*: Magnocellular Reticular Nucleus, *RM*: Nucleus Raphe Magnus, *RPa*: Nucleus Raphe Pallidus, *Gi*: Gigantocellular Reticular Nucleus, *LPGi*: Paragigantocellular Reticular Nucleus, lateral part.

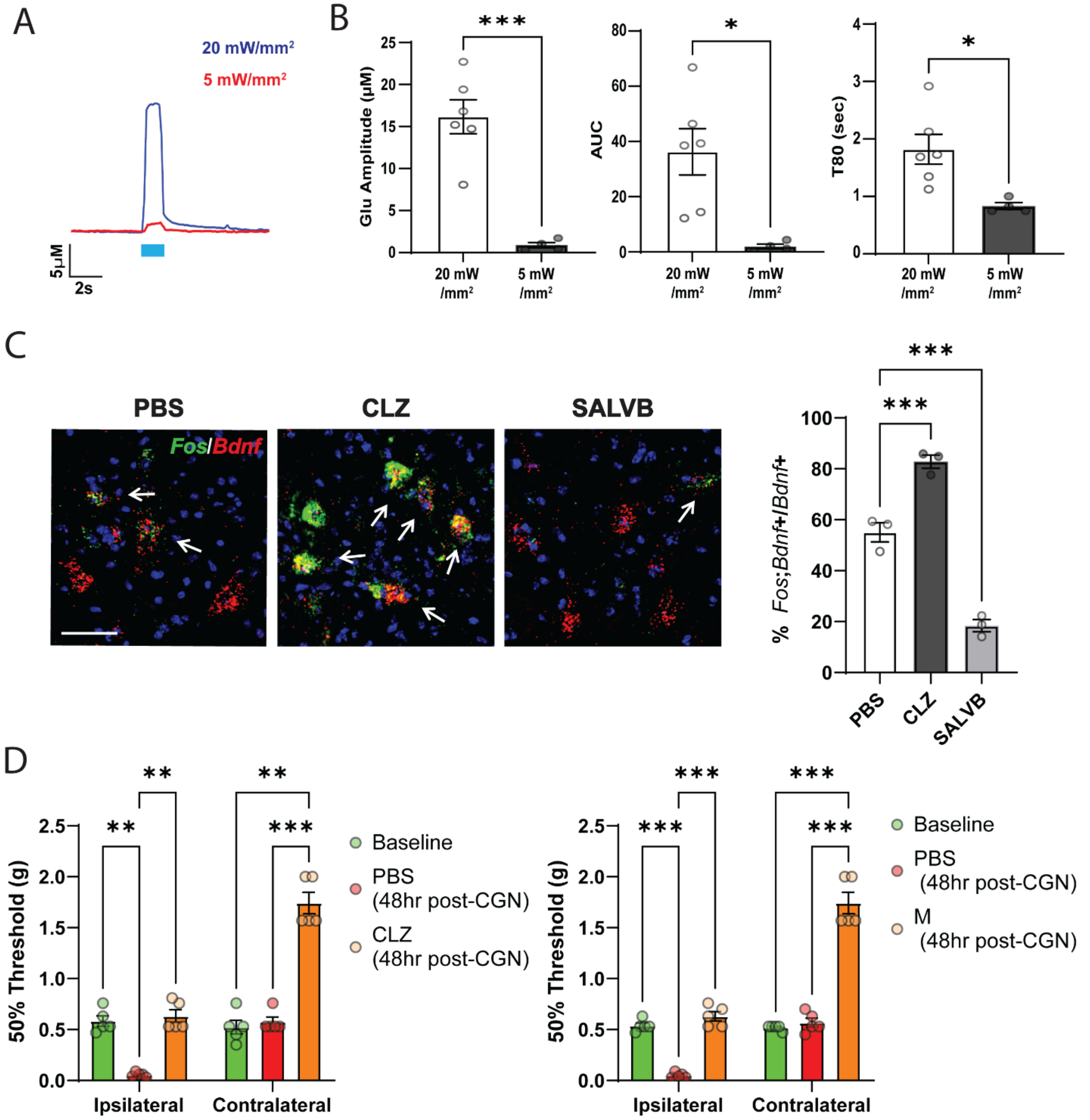

Figure S7: Glutamate release, Fos expression and attenuation of inflammatory allodynia by RVM^{BDNF} neurons (related to Fig. 4).

A, B) Two different LED intensity were used to test light-dependence of FAST-measured glutamate release in the spinal cord of mice with oChIEF-expressed in RVM^{BDNF} neurons. **A)** Representative traces of LED-induced glutamate amplitudes in the lumbar spinal cord as measured by FAST. Two different LED strengths were used: 20mW/mm^2 (blue) and 5mW/mm^2 (red). **B)** Assessment of the kinetics of light-evoked glutamate release. Glutamate amplitude (*left*), total Area Under the Curve (AUC, *center*), and time to 80% decay from peak amplitude

(T80, *right*) were quantified in response to either 20mW/mm^2 or 5mW/mm^2 blue light pulses. n ≥ 4 mice per condition, * $p < 0.05$, *** $p < 0.001$. **C)** BDNF-2A-Cre mice co-injected intracranially with Cre-dependent AAVs encoding hM3D(Gq) and hKORD were treated with either PBS, CLZ, or SALVB prior to sacrifice. *Left*, images from the RVM following fluorescence *in situ* hybridization for *Fos* (green) and *Bdnf* (red). Scale bar = 50µm. *Right*, Quantification of the proportion of *Bdnf*-expressing cells which also express *Fos*. n = 3 mice per condition, *** $p < 0.001$. **D)** As in Fig. 4K, mice were injected with intraplantar carrageenan (CGN), and 48 hours later von Frey threshold was assessed. Mechanical withdrawal thresholds were measured at baseline, 48 hours following carrageenan injection, and following activation of RVM^{BDNF} neurons (CLZ) or morphine (M). n = 5 mice per condition, ** $p < 0.01$, *** $p < 0.001$.

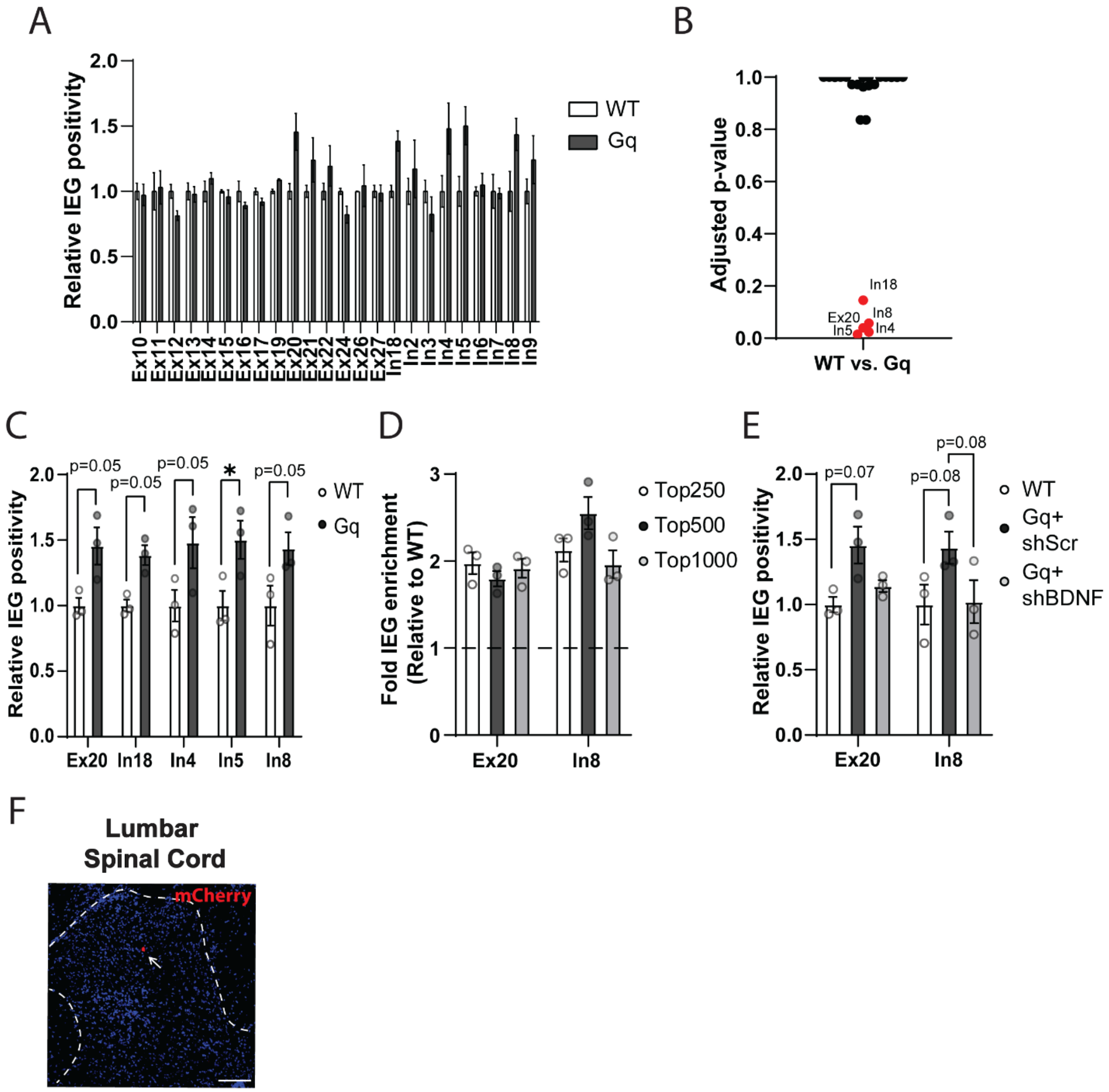

Figure S8: Additional data used for *in silico* analyses and *in vivo* validation of spinal targets of RVM^{BDNF} neurons (related to Fig. 5).

A-C) Assessment of IEG positivity was calculated for each cluster. IEG positivity was calculated as described in the Methods section. **A)** Relative IEG positivities for each cluster as compared between control mice treated with CLZ (WT), and mice with chemogenetic activation of RVM^{BDNF} neurons (Gq). n = 3 mice per condition. **B)** Adjusted p-values for the cluster-specific differences in relative IEG positivity observed in (**A**) between WT and Gq animals. The five clusters which showed significant or near-significant differences (Ex20, In18, In4, In5, and In8) are displayed in red. **C)** Relative IEG positivities for clusters Ex20, In18, In4, In5, and In8. n = 3 mice per condition, * $p < 0.05$. **D)** Enrichment analysis of spinal neurons isolated from animals

illustrated in Fig. 5A. Briefly, a list of the 250, 500, and 1000 most variable genes for each nucleus were compiled (Top250, Top500, and Top1000, respectively) and the immediate early genes present within those lists were compared to the remaining variable genes. Clusters which showed enhanced IEG expression relative to WT in all three analyses are displayed. n = 3 mice per condition. **E)** Relative IEG positivities for clusters Ex20 and In8 comparing control mice treated with CLZ (WT), mice with chemogenetic activation of RVM^{BDNF} neurons and scrambled control shRNA (Gq + shScr), and mice with activation of RVM^{BDNF} neurons and knockdown of BDNF expression using a shRNA (Gq + shBDNF). n = 3 mice per condition. **F)** Image from the lumbar spinal cord of a Gal-Cre mouse in which Cre-dependent TVA-oG-mCherry had been systemically injected and EnvA-pseudotyped Rabies virus expressing eGFP had been injected into the lumbar spinal cord. Sparse labelling of TVA-oG-mCherry-expressing cells (red) was observed in the dorsal horn. The white hatched line indicates the border of the dorsal horn. Arrow denotes a positive cell. Scale bar = 100µm.

Supplementary Statistics file
Statistical methods, including experimental groups, values for N, sex, parameters, statistical tests, and results for each experiment.
